# Structure and Assembly of TP901-1 Virion Unveiled by Mutagenesis

**DOI:** 10.1371/journal.pone.0131676

**Published:** 2015-07-06

**Authors:** Stephen R. Stockdale, Barry Collins, Silvia Spinelli, François P. Douillard, Jennifer Mahony, Christian Cambillau, Douwe van Sinderen

**Affiliations:** 1 School of Microbiology, University College Cork, Western Road, Cork, Ireland; 2 Aix-Marseille Université, Architecture et Fonction des Macromolécules Biologiques, Campus de Luminy, Case 932, 13288 Marseille Cedex 09, France; 3 Centre National de la Recherche Scientifique, AFMB, Campus de Luminy, Case 932, 13288 Marseille Cedex 09, France; 4 Alimentary Pharmabiotic Centre, University College Cork, Western Road, Cork, Ireland; Meharry Medical College, UNITED STATES

## Abstract

Bacteriophages of the *Siphoviridae* family represent the most abundant viral morphology in the biosphere, yet many molecular aspects of their virion structure, assembly and associated functions remain to be unveiled. In this study, we present a comprehensive mutational and molecular analysis of the temperate *Lactococcus lactis*-infecting phage TP901-1. Fourteen mutations located within the structural module of TP901-1 were created; twelve mutations were designed to prevent full length translation of putative proteins by non-sense mutations, while two additional mutations caused aberrant protein production. Electron microscopy and Western blot analysis of mutant virion preparations, as well as *in vitro* assembly of phage mutant combinations, revealed the essential nature of many of the corresponding gene products and provided information on their biological function(s). Based on the information obtained, we propose a functional and assembly model of the TP901-1 *Siphoviridae* virion.

## Introduction

*Myoviridae*, *Podoviridae* and *Siphoviridae* comprise the three familial divisions of *Caudovirales*, or tailed phages. *Siphoviridae* phages represent the dominant viral morphology, frequently outnumbering other viral morphotypes in environments such as the human gut and oceanic waters [1–4]. Despite the prevalence of phages with long non-contractile tails, only a handful of *Siphoviridae* have been extensively studied at structural and functional levels (for a review, see [5]). The prototypical and related *Lactococcus lactis*-infecting phages TP901-1 and Tuc2009 have been important in understanding *Siphoviridae* phages due to the genetic accessibility of their Gram-positive hosts, the various molecular tools available for manipulating their genomes and the conserved nature of phage structural proteins [6–13].

Recently, a composite model of the TP901-1 virion was constructed by single-particle electron microscopy (EM) [14]. In addition, several studies have focused on characterizing the structure and function of lactococcal phage baseplates, as these tail-tip structures possess the receptor-binding proteins that determine the specific recognition of and initial interactions with their particular host(s) [15–25]. While the capsid, head-tail connector, and tail-tube assemblages of TP901-1 and Tuc2009 phages have not been described at atomic resolution, the EM-derived ~20Å resolution structure of phage TP901-1 [14] illustrates the common features shared with other lactococcal [26] and non-lactococcal *Siphoviridae* [27]. Therefore, valid predictions can be made regarding these regions due to the evolutionary conserved nature of tailed phages and their structural proteins [28].

Several *Caudovirales* phages, such as P22, ϕ29 and HK97, have served as models for understanding the structure and assembly of phage capsids [29, 30], while particular *Siphoviridae*, including phages λ, T5, SPP1 and p2, have been instrumental in assessing the assembly and role of the tail during infection [26, 31–33]. Aksyuk and Rossmann (2011) have reported that the morphogenesis of many icosahedral phage capsids require at least three essential factors: the portal protein, the major head protein (MHP), and a scaffolding protein, or a scaffolding domain associated with the MHP [5]. The assembly of functional capsids, through an intermediate DNA-free procapsid structure, necessitates MHP subunits to assemble into pentamers and a variable number of hexamers, and incorporate a dodecameric portal protein complex into a unique capsid vertex [34]. While scaffolding proteins or domains are not believed to be part of mature capsid structures [35], portal protein complexes and scaffolding proteins have been demonstrated to interact and are proposed to initiate capsid morphogenesis [36–38]. In a model of phage P22 capsid assembly, Prevelige and colleagues (1993) visualized incomplete capsid structures consistent with a model whereby a single portal vertex nucleates capsid morphogenesis, and where scaffolding proteins and MHPs are sequentially added to the edges of the assembling procapsid shell [39, 40]. Head-tail connector proteins of *Siphoviridae* phages, such as HK97 and SPP1, associate with the portal protein-containing vertex of DNA-packaged capsids and serve as an attachment site for the tail organelle [41–43].

*Siphoviridae* tail assembly requires the tail tape measure protein (TMP). For phages λ, T5 and SPP1, the TMP is predicted to exist as a hexameric complex which dictates the length of the phage tail [6, 44–47]. Two tail assembly chaperone (TAC) proteins, gpG and gpGT, which have been extensively studied using phage λ as a model, are essential for the correct assembly of *Siphoviridae* tails. Chaperone gpGT is produced through a -1 ribosomal frame-shift during mRNA translation at a specific ‘slippery’ sequence corresponding to the 3’ end of gene *gpG*, representing a genetic peculiarity conserved in many dsDNA tailed phages [48]. Protein gpGT is believed to bind to the hydrophobic TMP using its G domain while recruiting the phage major tail protein (MTP) via its T domain [49, 50]. Polymerization of MTP around and along the TMP to form the phage tail tube pauses when the end of the TMP is reached [51]. Tail terminator proteins associate with the terminal hexameric ring of the *Siphoviridae* tail tube to complete MTP polymerization [52–55]. In phage λ, tails also require activation through an unknown mechanism by protein gpZ before they can associate with phage capsids to produce complete virions [51].

In the current study, we analysed the effect of fourteen mutations introduced into genes *orf32*-*44* of the structural module of TP901-1. We examined the efficiency of plaquing and phage virion integrity, as determined by *in vitro* assembly, immunological detection and electron microscopy. This new data complements and significantly expands current knowledge on lactococcal *Siphoviridae* [6, 9, 56], allowing the formulation of a detailed molecular model describing the structure, and the hierarchical function and assembly of the TP901-1 virion components.

## Materials and Methods

### Bioinformatic Analysis

DNA sequences were downloaded from NCBI GenBank [57]. Inducible prophage t712 of NZ9000 [58], called TP712 by Roces *et al*. (2013) [59], was identified using the PHAge Search Tool (PHAST) [60]. BLAST, Pfam and HHpred analyses were used for functional annotations of proteins [61–64]. Putative promoter sequences of NZ9000 prophage t712 were identified using SoftBerry BPROM (http://www.softberry.com). Significant HHpred hits of TP901-1 structural module proteins are presented in Table 1, alongside Protein Data Bank (PDB) identification codes and the original source of the hit. Alignment of multiple amino acid sequences was performed using ClustalW [65]. Protein secondary structure predictions and intrinsically disordered regions were detected using Quick2D (http://toolkit.tuebingen.mpg.de/) [66–70].

**Table 1:** Bioinformatic analysis of TP901-1’s predicted structural-encoding proteins.

Orf	Start	Stop	Size (aa)	Mw (kDa)	pI	Putative function	Abbreviation	HHpred	Function
30	13562	13972	136	15.44	8.1	Terminase small subunit	TerS	100; 3ZQP; SPP1	Terminase small subunit
31	13965	15353	462	53.10	6.6	Terminase large subunit	TerL	100; 4BIJ; T7	Terminase large subunit
32	15354	16712	452	51.82	4.4	Portal protein	Portal	100; 2JES; SPP1	Portal protein
33	16716	18410	564	64.28	9.1	-	MCP1	n.s.h.	-
34	18425	18652	75	9.27	6.4	-	MCP2	n.s.h.	-
35	18767	19429	220	24.54	4.6	Scaffolding protein	Sfp	n.s.h.	Scaffolding protein
36	19431	20249	272	28.74	5.1	Major head protein	MHP	100; 4AN5; SPP1	Major head protein
37	20249	20446	65	7.50	9.6	-	MCP3	98.5; 2OUT; Mu-like	-
38	20433	20765	110	12.83	4.6	Head-tail connector	HTC1	99.4; 2KBZ; SPP1gp15	Head-tail connector
39	20762	21073	103	12.18	4.9	Head-tail connector	HTC2	94.5; 2KCA; SPP1 gp16	Head-tail connector
40	21070	21408	112	12.46	9.6	-	Tap	n.s.h.	-
41	21405	21794	129	14.76	7.2	Tail terminator protein	Ttp	100; 2LFP; SPP1 gp17	Tail terminator
42	21805	22314	169	18.63	4.7	Major tail protein	MTP	99.3; 2K4Q; Lambda gpV	Major tail protein
43	22429	22764	111	12.47	4.9	Chaperone	gpG	n.s.h.	-
44	22803	23123	130	15.53	8.6	Chaperone	gpT	n.s.h	-
45	23138	25951	937	100.28	8.8	Tape measure protein	TMP	89; 3R2P;Helixbundle	Tape measure protein
46	25961	26722	253	29.13	5.6	Distal tail protein	Dit	X-ray;4DIV	Baseplate, Dit
47	26722	29478	918	102.07	5.4	Tail-associated lysin	TalNter	100; 3GS9; Prophage gp18	T4 gp27-like
						Tail-associated lysin	Tal Cter	100; 3CSQ; Hydrolase	Zn peptidase
48	29491	30390	299	33.84	5.1	Upper baseplate protein	BppU	X-ray; 4DIV	Baseplate, BppU
49	30405	30896	163	17.15	7.9	Lower baseplate protein	BppL	X-ray; 4DIV	Baseplate, BppL or RBP
50	30910	31134	74	8.65	6.7	-	Hypothetical	94.2; 3HD7; Human	Membrane associated?
51	31147	33150	667	71.57	4.9	Neck passage structure	NPSNter	98; 4DIV; TP901-1	ORF48 Nter-like
						Neck passage structure	NPSCter	95; 2XOM; Hydrolase	Glucosidehydrolase

Genomic start and stop coordinates of structural module encoding genes are derived from GenBank accession number NC_002747. The percentage similarity of TP901-1 protein sequences to solved protein structures using HHpred, including the source of the homologue, is shown. Several TP901-1 proteins had no significant homologue (n.s.h.) detected.

### Bacteria, Phages and Growth Conditions

Bacterial strains and phages used in this study are listed in S1 Table. Growth experiments of lactococcal strains and phage propagations were performed as described previously [13, 71]. TP901-1*erm* and mutant derivatives were induced from corresponding lysogens of *L*. *lactis* NZ9000-Cro_t712_ using the following conditions: the relevant strains were grown at 30°C to an *A*_600_ of 0.3, at which point mitomycin C (Sigma) was added to a final concentration of 3 μg/ml with subsequent incubation at 20°C until lysis occurred. NaCl, 1 M (w/v) final concentration, was added to the resultant phage lysates followed by centrifugation in a Thermo Scientific SL16R centrifuge at 5580 × *g* for 15 min and stored at 4^°^C.

### Mutant Generation

Recombineering mutagenesis was performed as described previously [13, 72, 73]. A detailed description of all TP901-1*erm*-derived mutants created in this study is provided in S2 Table. Point mutations, creating a BamHI restriction site, were introduced in the deduced promoter sequence of the predicted anti-repressor-encoding gene (*cro*_t712_) of prophage t712 of NZ9000 in order to prevent its induction during mitomycin C addition (generating strain NZ9000-Cro_t712_). Mutations in targeted ORFs of the TP901-1 structural module were generated so as to insert an in-frame stop codon, thereby terminating translation (mutant nomenclature ORF_TP901-1_::Ter), and where possible, introduce a restriction enzyme site. In order to prevent gpT production, an in-frame BamHI restriction site was introduced into the ‘slippery’ sequence (GGGAAAG) at the 3’ end of the TP901-1 *gpG* gene which is required for the sequence-specific -1 ribosomal frame-shift required for gpT translation (mutant termed gpT_TP901-1_::BamHI) [48]. To further analyse the production and role of chaperone protein gpT, a single nucleotide insertion, coupled with several point mutations, was introduced into the *gpG* ‘slippery’ sequence resulting in a direct translational fusion between the *gpG* and *gpT* gene sequences (the resulting mutant phage was designated gpG*fs*T_TP901-1_). Mismatch Amplification Mutation Assay (MAMA) PCRs using oligonucleotides complementary at their 3’ end to the desired nucleotide changes [73], and restriction digests, were performed to identify NZ9000-Cro_t712_ derivatives (following recombineering mutagenesis) which contained the anticipated mutated TP901-1*erm* prophage. Oligonucleotides used for recombineering and MAMA PCR screening reactions were purchased from Integrated DNA Technologies (IDT, Belgium), and are listed in S3 Table. Mutations were confirmed by Sanger sequencing relevant PCR-amplified regions using chromosomal DNA from the mutated TP901-1*erm*-containing *L*. *lactis* NZ9000-Cro_t712_ lysogen as a template (sequencing performed by MWG, Germany).

### Phage Purification

Phage lysates of TP901-1*erm* wild type and the TP901-1*erm*-derived mutants Portal_TP901-1_::Ter, MCP1_TP901-1_::Ter, MHP_TP901-1_::Ter, Ttp_TP901-1_::Ter, MTP_TP901-1_::Ter, gpG_TP901-1_::Ter, gpT_TP901-1_::BamHI and gpG*fs*T_TP901-1_ were purified by sucrose gradient ultracentrifugation as follows: polyethylene glycol 8000 (PEG8000; 10% w/v) was added to NaCl-treated (as described above) phage lysates and mixed gently at Room Temperature (RT) until completely dissolved. Phage samples were placed on ice for 3–4 hr before centrifugation in a 4°C pre-chilled Thermo Scientific SL16R centrifuge at 5580 × *g* for 20 min. Pellets were air-dried, before resuspending in 1:50 initial volume of SM buffer (50 mM Tris-HCl, 100 mM NaCl, 10 mM MgSO_4_; pH 7.5). PEG8000 was removed from phage samples by extraction with an equal volume of chloroform, followed by vortexing for 30 sec and centrifugation at 1660 × *g* for 15 min. The aqueous phase was extracted and applied to a sucrose gradient. Linear sucrose gradients, 20–70% (w/v), were prepared using a Masterflex peristaltic pump (Thermo Scientific) in Beckman Thinwall Ultra-Clear centrifuge tubes (14 x 89 mm). Sucrose was added to all phage samples, at a final concentration of 14% (w/v), before layering them on top of the prepared linear sucrose gradients. Samples were centrifuged for 1.5 hr at 250,000 × *g* in a Beckman SW40Ti ultracentrifuge rotor at 4°C. Bands were extracted from the sucrose gradient and immediately dialysed against SM buffer (12–14 kDa MW cut-off; Medicell International Ltd., London, UK). For TP901-1*erm*-derived mutants MCP2_TP901-1_::Ter, Sfp_TP901-1_::Ter, HTC1_TP901-1_::Ter, HTC2_TP901-1_::Ter and Tap_TP901-1_::Ter, CsCl purification was performed instead of sucrose gradient purification as this permitted higher quality visualization of the produced virion structures. Chloroform-extracts of PEG-precipitates of mutant phage lysates (as described above for sucrose gradient purification) were prepared and then used for CsCl gradient purification of phage particles as previously described [74].

### Phage Assays

Spot and plaque assays of TP901-1*erm* and its mutant derivatives were performed as described previously [75]. *In vitro* assembly of TP901-1*erm*-derived structural module mutants was performed using sucrose gradient-purified bands of TP901-1*erm* and mutant derivatives of this phage (in order to standardise the results obtained, only sucrose gradient-purified bands, and not CsCl purified bands, were tested). Sucrose gradient-purified fractions of mutant MHP_TP901-1_::Ter and mutant MTP_TP901-1_::Ter were used as either a phage tail or capsid donor, respectively. Briefly, 10 μl of purified TP901-1*erm* capsids or tails were mixed with 10 μl of purified TP901-1*erm* structural module mutant derivative fractions, and incubated for 16 hr at 30°C. The phage mix was then tested for its ability to infect *L*. *lactis* 3107 by assessing the frequency of lysogeny, which was determined through acquisition of the adenine methylase-encoding gene present on the TP901-1*erm* phage genome (or its mutant derivatives), conferring resistance to 5 μg/ml erythromycin [11, 76]. This was performed by adding 50 μl of the phage mix to 450 μl of *L*. *lactis* 3107 at an *A*_600_ of 0.3 and incubated at 30°C for 1 hr before plating. Three technical replicates calculating the frequency of lysogeny were performed and results averaged.

### Protein Expression, Purification and Antibody Generation

Antibodies against Tuc2009 tail proteins were previously generated and proved successful in detecting the homo-immune structural proteins of Tuc2009 and TP901-1 [10]. Therefore, the following Tuc2009 capsid proteins were overproduced and purified in *L*. *lactis* NZ9000: Portal_Tuc2009_, MCP1_Tuc2009_, MCP2_Tuc2009_, Sfp_Tuc2009_, MHP_Tuc2009_, MCP3_Tuc2009_, HTC1_Tuc2009_ and Tap_Tuc2009_. Attempts to express recombinant proteins of HTC2_Tuc2009_ and Ttp_Tuc2009_ were unsuccessful (data not shown). Recombinant protein-expression constructs created during this study, and the oligonucleotides used to create them, are outlined in S1 and S3 Tables, respectively. Primers used for cloning were purchased from Eurofins MWG (Germany). KOD high-fidelity DNA polymerase (Novagen, UK) was used for PCR amplifications. For cloning reactions, restriction enzymes were supplied by Roche (Germany) and ligations were performed with T4 DNA ligase (Promega, USA). For genes cloned using pNZ8048, NcoI and SpeI were used, while for pTX8049, BamHI and SpeI were employed. In both cases, primers were used so as to facilitate the incorporation of a hexahistidine-tag at the C-terminus of the encoded protein, while pTX8049-generated proteins also incorporate an N-terminally fused thioredoxin tag [77]. Proteins were expressed and purified as outlined previously [77]. Rabbit antibody production was carried out by Harlan Laboratories (Leicester, UK). Immunization was initially carried out with individual proteins supplemented with Freund’s adjuvant at a concentration of ~200 μg/ml; this was followed by five booster injections over the 112 day protocol.

### SDS-PAGE and Western Blotting

SDS-PAGE separation of proteins was performed using 12% or 15% (v/v) acrylamide gels following standard procedures [78]. After separation of ~10 μg protein by SDS-PAGE, proteins were transferred to a nitrocellulose membrane by electro-blotting using 10 mM CAPS [3-(cyclohexylamino)-1-propanesulfonic acid; pH 11], 10% methanol transfer buffer. Membranes were blocked for 1 hr at RT with 50% (v/v) Odyssey blocking buffer in PBS (Licor, USA). Optimal conditions for each rabbit polyclonal antibody were determined empirically and the reproducibility of the results verified by repeated Western blot analyses during these optimisations (data not shown). Membranes were incubated with primary rabbit polyclonal antibodies, in a range from 1:500–1:15000, in blocking buffer containing 0.12–0.25% (v/v) Tween-20 for 1 hr at RT or overnight at 4°C. Following 4 × 5 min washes in 0.1% (v/v) Tween-20, the membranes were incubated in 1:7500 fluorescently labelled goat anti-rabbit IgG secondary antibody (Licor, USA) in blocking buffer containing 0.12–0.25% Tween-20. Membranes were washed again and visualised on a Licor Odyssey imaging system (USA).

### Electron Microscopy

For negative staining, 5 μl of each sample were applied onto glow-discharged carbon-coated grids (Agar Scientific, Stansted, UK) and incubated for 1 min. The grids were washed with 5 μl of deionized water before incubating for 30 sec in 1% (w/v) of Uranyl Acetate (Agar Scientific, Stansted, UK). CCD images were collected using a Tecnai Spirit operated at 120 Kv and a 2Kx2K CCD camera.

## Results

### Optimizing TP901-1*erm* host background

Several improvements were made to optimize the host background for analysing mutations made in TP901-1*erm*, a derivative of TP901-1 that is marked with an erythromycin-resistance (Em^r^) cassette[76], which had been used to select a TP901-1*erm* lysogenized derivative of strain *L*. *lactis* NZ9000 [13]. This lysogenized NZ9000 derivative offered an advantage over the original TP901-1*erm* host, *L*. *lactis* 901–1, as the latter strain contains a second prophage similar in sequence to TP901-1 which may supply proteins *in trans* (Finn Vogensen, personal communication). In addition, NZ9000 contains an integrated copy of the NisRK cassette for protein expression using the nisin-inducible promoter system [79], which was used for the overproduction of RecT for recombineering mutagenesis [73]. In order to further optimize NZ9000 as a background for analysing mutations in TP901-1*erm*, a BamHI restriction site was introduced into the deduced -10 promoter sequence of the lysogenic/lytic switch of prophage t712 (thereby generating strain NZ9000-Cro_t712_). Causing ablation of this promoter was aimed at preventing t712 prophage induction and virion production. Induction of prophage t712 was identified as a potential problem as; (i) t712 particle production would interfere with TP901-1 phage particle study, (ii) t712 induction would increase the metabolic burden on the host when only TP901-1 induction was desired, and (iii) it was deemed possible that, due to the conserved nature of *Siphoviridae* proteins, t712 may supply TP901-1 with structural proteins being analysed during this study *in trans* [59]. This expectation was indeed verified, as mutagenesis of the deduced promoter sequence for t712 Cro protein caused an approximate 100-fold increase in the number of produced infective TP901-1*erm* phage particles obtained following mitomycin C induction from the NZ9000-Cro_t712_ background as compared to that obtained when strain NZ9000 was used (data not shown; indicator strain used for TP901-1*erm* phage titre determination was *L*. *lactis* 3107).

### Annotation of TP901-1*erm* Structural Module and Mutants

**Fig 1:**
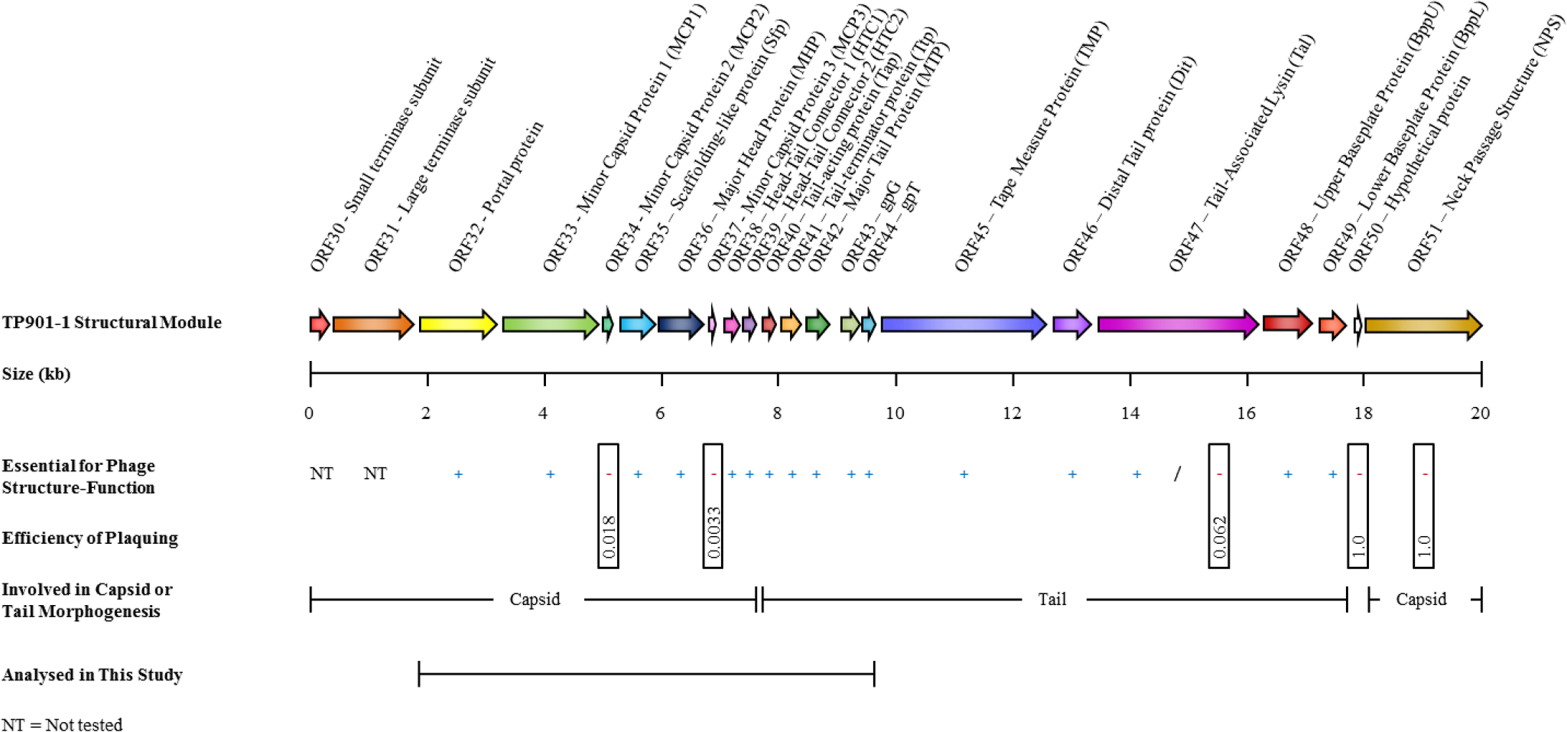
Schematic representation of the 22 annotated genes of the structural module of phage TP901-1. Mutagenesis of individual genes of the TP901-1 structural module showed gene products to be essential (+) or non-essential (-) for creating infectious phage virions. The efficiency of plaquing is displayed for mutants of non-essential gene products. The genomic regions encoding capsid and tail morphogenesis proteins are indicated and such assignments are based on data described in the text.

The structural module of TP901-1 is represented by a polycistronic operon, approximately 20 kb in length, transcribed from a promoter located upstream of the gene encoding the predicted small terminase subunit [7, 8, 12]. This structural module of TP901-1 is predicted to encode 22 proteins (Fig 1) [7]. Functional analyses have previously been performed for a number of TP901-1 structural proteins (and by inference those of the closely related phage Tuc2009) [6, 9, 11, 13, 16, 19, 56, 80, 81]. Due to the conserved nature of phage structural proteins, functions of several TP901-1 proteins can be predicted based on a number of well characterized phage prototypes [42, 55, 82–84]. However, some TP901-1 proteins do not exhibit significant similarity to known proteins and thus their functional assignment is based on literature and bioinformatic analyses.

Stop codon mutations in TP901-1*erm* structural module mutants were created, via recombineering, in the identified genes from *orf32* through to *orf43*, inclusive, which encode the portal protein and the TAC gpG, respectively. Two additional mutations were created to investigate the production and role of *orf44*, encoding gpT (S2 Table). The nomenclature assigned to TP901-1*erm* structural module mutants is based on the abbreviated name assigned to the phage open reading frames (Table 1), followed by a designation signifying the insertion of a translation terminating stop codon or restriction site (e.g. MCP1_TP901-1_::Ter or gpT_TP901-1_::BamHI, respectively); the TP901-1*erm* mutant that carries a translational fusion mutation of ORF *gpG* and *gpT* was termed gpG*fs*T_TP901-1_ (see also Materials and Methods section).

### Complementation of TP901-1*erm* Structural Mutants

Individual TP901-1*erm*-derived mutants were induced from their lysogenic host by mitomycin C (see Materials and Methods). Obtained lysates were then tested for their ability to infect host strain *L*. *lactis* 3107 by spot and plaque assays to determine which genes/proteins are essential for TP901-1 phage assembly and/or infection. Of the 14 mutants created in the TP901-1*erm* structural module, only mutants carrying a non-sense mutation in the genes for MCP2_TP901-1_ or MCP3_TP901-1_ retained sufficient residual infectious ability to form plaques, while at the same time displaying decreased Efficiencies of Plaquing (EOPs) relative to wild-type TP901-1*erm* control (*p*-values < 0.01; Fig 1). Mutant phages were then tested for the ability to lysogenize their host bacterium. Lysogeny assays, which reflect a phage’s ability to adsorb to its host and to perform DNA injection followed by integration, rather than its ability to form a plaque, demonstrated that sucrose gradient-purified preparations of mutants MCP1_TP901-1_::Ter, Sfp_TP901-1_::Ter, HTC1_TP901-1_::Ter, HTC2_TP901-1_::Ter and Tap_TP901-1_::Ter were able to generate Em^r^ lactococcal colonies (Table 2). While lysogeny frequencies of these mutants were substantially below that of the TP901-1*erm* control, the results were noted in subsequent analyses.

**Table 2:** Lysogeny by TP901-1*erm* and structural module purified mutants mixed with phage capsids and tails.

Sample	Lysogeny by Purified Sample^1^		
	Purified Sample	Mixed with Phage Capsids	Mixed with Phage Tails
**TP901-1*erm***	7.51e06 +/- 4.25e05	NT	NT
**Portal_TP901-1_::Ter**	0.00	5.90e04 +/- 4.36e03	0.00
**MCP1_TP901-1_::Ter**	2.70e02 +/- 3.00e01	7.80e02 +/- 8.72e01	1.33e02 +/- 1.53e01
**MCP2_TP901-1_::Ter**	6.52e06 +/-3.07e05	NT	NT
**Sfp_TP901-1_::Ter**	6.00e01 +/-6.93e01	5.43e04 +/-1.57e04	5.33e01 +/-2.31e01
**MHP_TP901-1_::Ter**	0.00	2.30e05 +/-5.01e04	0.00
**MCP3_TP901-1_::Ter**	5.05e06 +/-9.1e05	NT	NT
**HTC1_TP901-1_::Ter**	2.62e04 ^+^/-1.88e03	9.53e04 ^+^/-2.48e04	1.38e04 ^+^/-2.75e03
**HTC2_TP901-1_::Ter**	7.33e01 ^+^/-5.51e01	1.43e05 ^+^/-2.55e04	2.00e01 ^+^/-2.00e01
**Tap_TP901-1_::Ter**	4.87e02 ^+^/-1.02e02	1.97e02 ^+^/-4.73e01	2.46e05 ^+^/-2.01e04
**Ttp_TP901-1_::Ter**	0.00	0.00	2.78e05 ^+^/-2.96e04
**MTP_TP901-1_::Ter**	0.00	0.00	2.01e05 ^+^/-2.98e04
**gpG_TP901-1_::Ter**	0.00	0.00	2.54e05 ^+^/-3.80e04
**gpT_TP901-1_::Ter**	0.00	0.00	1.30e05 ^+^/-2.41e04
**gpG*fs*T_TP901-1_**	0.00	0.00	0.00

Lysogeny of purified samples, either by purified sample on their own or mixed with phage capsids or tails, resulting in Em^r^ *L*. *lactis* 3107 colonies. The lack of Em^r^ colonies following lysogeny tests by gpG*fs*T_TP901-1_ on its own or mixed with capsids or tails suggests that tail tube polymerization is affected.

^1^ *L*. *lactis* 3107 Em^r^ cfu/ml; NT = Not tested

Previous studies of phage λ have shown that capsids and tails can be produced independently, and, following purification and subsequent mixing, can assemble spontaneously *in vitro* [85]. In order to determine if capsid and tail structures of lactococcal phage TP901-1 are similarly capable of *in vitro* assembly, purified capsids and tails of TP901-1 were complemented with the generated mutants and tested for the ability to lysogenize host strain *L*. *lactis* 3107. Unable to establish lysogeny on their own, following incubation of purified MHP_TP901-1_::Ter and MTP_TP901-1_::Ter, which act as intact phage tail and capsid donors, respectively, lysogenization of *L*. *lactis* 3107 did occur as was deduced from the appearance of Em^r^-colonies (Table 2). This demonstrates that lactococcal phage TP901-1*erm* capsids and tails can indeed assemble *in vitro* to form functional, DNA-injecting virions.

By complementing TP901-1*erm* structural module mutants through the addition of either functional capsids or tails, it was possible to distinguish gene products required for functional capsid formation from those that are crucial for tail production. There is a clear distinction between TP901-1*erm* mutants that establish lysogeny at higher levels following *in vitro* assembly with either phage capsids or phage tails. TP901-1*erm* derivatives carrying individual mutations targeting the portal protein-encoding gene (*orf32*) through to the gene specifying the HTC2 protein (*orf39*) were able to lysogenize *L*. *lactis* 3107 following incubation with purified capsids (donated from TP901-1*erm* MTP mutant; see Materials and Methods section) to a significantly higher level as compared to the mutant on its own or when mixed with purified tails (Table 2). An exception to the *in vitro* assembly and lysogeny assays is MCP1_TP901-1_::Ter, where only a slight increase in lysogeny frequency is observed when purified capsids are added to the mutant preparation. This suggests that capsids and tails of the MCP1_TP901-1_::Ter mutant do connect during virion assembly but that the assembled structure is largely defective in achieving infection (see Discussion section). TP901-1*erm* mutations in genes *tap* (*orf40*) to *gpT* (*orf44*) were significantly increased in their frequency of lysogeny following the addition of purified tails compared to lysogeny by the purified samples on their own or mixed with purified capsids. In contrast, the frequency of lysogeny of mutant gpG*fs*T_TP901-1_ did not increase following the addition of purified capsids or tails (discussed below).

### Western Blot and Electron Microscopic Analysis of TP901-1*erm* Structural Mutants

**Fig 2:**
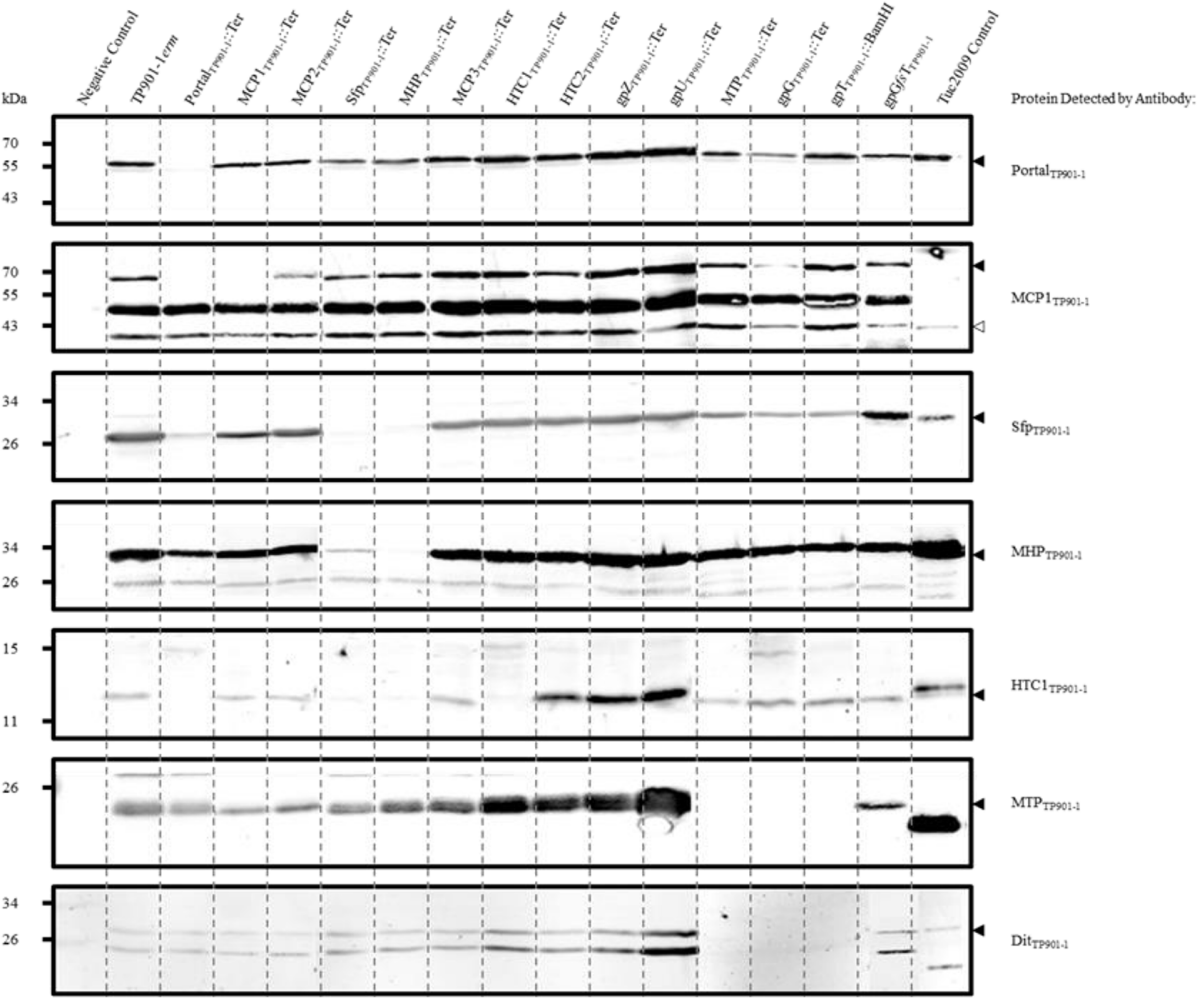
Immunological detection of TP901-1*erm* structural proteins precipitated from phage lysates by PEG8000. Negative control represents PEG8000 precipitation of mitomycin C induced strain NZ9000-Cro_t712_. The protein detected by the primary antibody is depicted at the right hand side of the image, with black-filled triangles highlighting the TP901-1 protein sizes and a black-unfilled triangle indicating the MCP1_Tuc2009_ protein in the Tuc2009 control lane that is ~25 kDa smaller than MCP1_TP901-1_. Western blots against MCP1_TP901-1_ resulted in two non-specific bands, at approximately 50 and 40 kDa, that could not be removed during optimisation.

**Fig 3:**
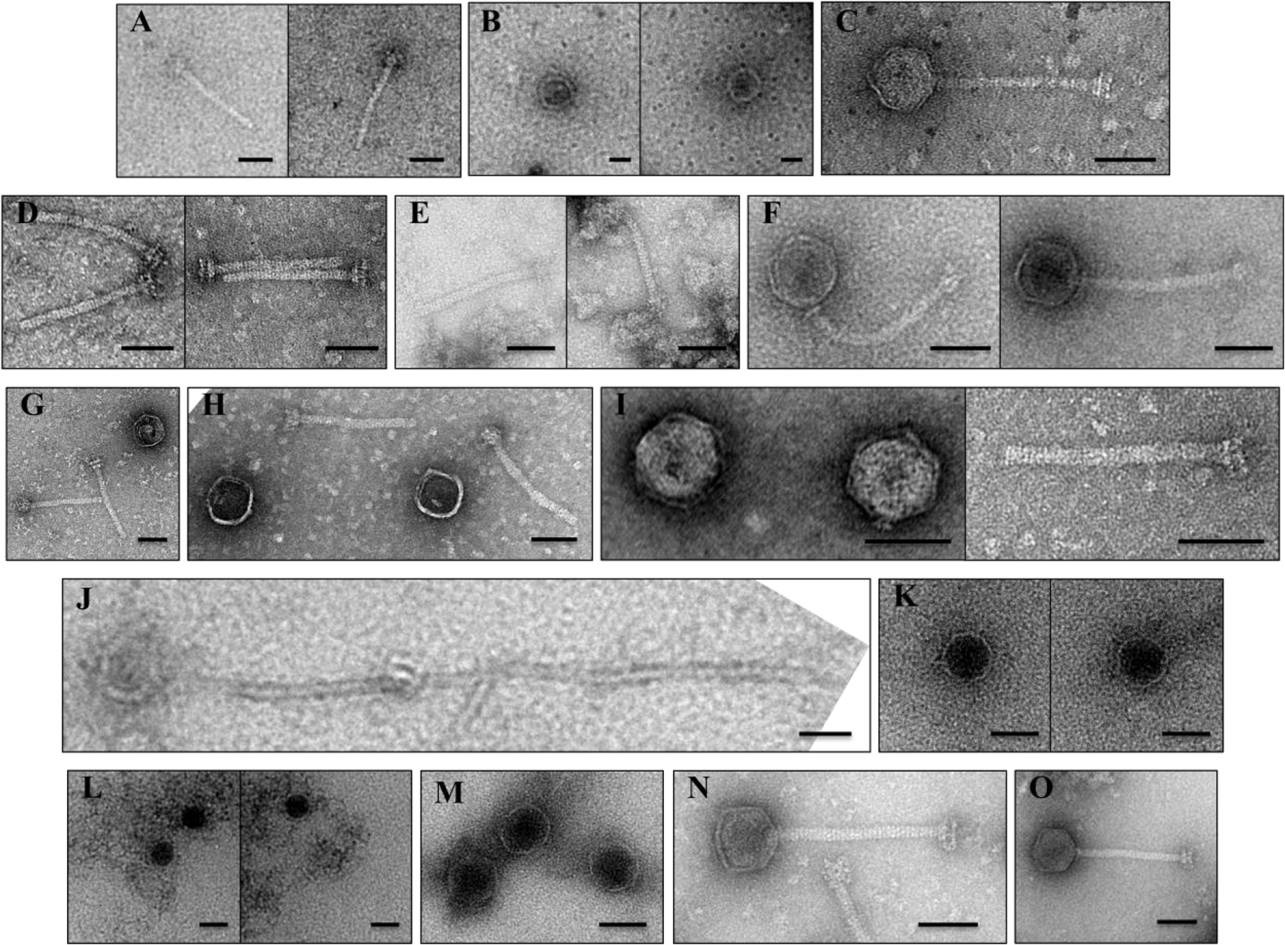
EM images of (A-N) TP901-1*erm* structural module mutants and (O) TP901-1*erm* control. (A) Few tails, but no capsids, produced by mutant Portal_TP901-1_::Ter. (B) Capsids, without attached tails, visualised for mutant MCP1_TP901-1_::Ter. (C) Full, but scarce, phage particles produced following mutation MCP2_TP901-1_::Ter. (D) Tails, but no capsids, produced for mutant Sfp_TP901-1_::Ter. (E) Tails-only produced for mutant MHP_TP901-1_::Ter. (F) Mature-resembling phages produced for mutant MCP3_TP901-1_::Ter. (G) Unconnected capsids and tails observed in HTC1_TP901-1_::Ter. (H) Distinct capsids and tails observed in HTC2_TP901-1_::Ter. (I) Separated capsids and tails imaged from mutant Tap_TP901-1_::Ter. (J) Capsids with long polytails isolated for mutant Ttp_TP901-1_::Ter. (K) Capsids only are observed for mutant MTP_TP901-1_::Ter. (L) Capsids and insoluble aggregates, proposed to be the TMP, observed in preparations of mutant gpG_TP901-1_::Ter. (M) Capsids, but no tails, observed in preparations of mutant gpT_TP901-1_::BamHI. (N) Mature-resembling phage produced by mutant gpG*fs*T_TP901-1_. (O) Control TP901-1*erm* mature infectious phages. Refer to text for a more detailed description characterizing each mutant. Scale bars are 50 nm long.

In order to further assess the functionality of the mutant derivatives of TP901-1, and to ascertain if negative polar downstream effects were the cause of loss/reduction of infectivity of these mutants, a dual approach of Western blotting (employing antibodies raised against capsid and tail-associated proteins) and electron microscopic (EM) analysis of sucrose or CsCl gradient purified particles was applied (Figs 2 and 3, respectively). EM analysis of purified phage lysates of TP901-1*erm* mutants was performed to visualize structural assemblages. For each phage mutant, approximately 50 particles were observed to establish the reproducibility of the analysis. Through these observations, complete phage particles or separated head (DNA-filled or empty) and tail structures were discerned, depending on the mutant.

In the majority of cases, both Western blotting and EM analysis indicated that the introduction of capsid mutations did not affect the production of many downstream capsid-related functions, and also that tails were produced for the vast majority of cases. This would indicate that in most instances, polar effects were not the cause of loss/reduced infectivity of these mutants. However, there are some exceptions, which are discussed below, and in these cases, other capsid-related proteins appear not to be produced, while downstream-encoded tail structures are still being produced and assembled. It is possible that the capsid-related proteins are absent because they are required for complex formation with the mutated element and are therefore lost during the purification process,or their expression is negatively affected due to polar effects of the introduced mutation, such as those associated with translational coupling or translational fusion.

Immunoblot detection was performed on chloroform-treated PEG8000-mediated precipitates of induced TP901-1*erm* mutants to determine the role of the different proteins during virion assembly (Fig 2). Analysis of PEG-precipitated protein fractions was performed as; (i) direct analysis of phage lysates was not sensitive enough, (ii) analysis of PEG-precipitated phage lysates was predicted to be more accurate and reproducible than samples prepared by ultracentrifugation, (iii) PEG is anticipated to precipitate only large proteins or proteinaceous complexes such as those which form during phage assembly, and (iv) it was anticipated that some phage proteins and (small) protein assemblies would be lost during the preparation and centrifugal forces generated during purification procedures. However, while several potential protein-protein interactions are inferred during the analysis of PEG-precipitated lysates, due to the complex nature of phage assembly, these observations will require further experimental scrutiny to validate their roles in our proposed phage assembly model. Immunoblot detection of the portal protein of TP901-1 demonstrated it is detectable in all TP901-1*erm* mutants, and the purified phage controls, except for the lysate obtained from the TP901-1*erm* mutant that carries a non-sense mutation in the gene encoding the portal protein (Fig 2). This result validates the recombineering mutagenesis strategy, confirming that the insertion of an in-frame stop codon successfully prevents its production. As expected, EM images of purified fractions of Portal_TP901-1_::Ter showed only tail structures produced, and no detectable capsid structures (Fig 3A). In addition to the absence of the portal protein in PEG-mediated precipitates of mutant Portal_TP901-1_::Ter, proteins MCP1_TP901-1_ and HTC1_TP901-1_ are also undetectable in Western blots of Portal_TP901-1_::Ter. Also, while not quantified, there appears to be a decreased amount of proteins Sfp_TP901-1_ and MHP_TP901-1_ in Western blots of mutant Portal_TP901-1_::Ter. The decreased detection/loss of these proteins in immunoblots of mutant Portal_TP901-1_::Ter indicates that they associate by specific protein-protein interactions during TP901-1 virion assembly, although this presumption will require experimental validation (see Discussion).

As expected, MCP1_TP901-1_ was not observed in Western blots using purified preparations of mutant MCP1_TP901-1_::Ter. In addition, no noticeable effect was caused by this mutation in any of the additional proteins tested by Western blot, indicating that downstream polar effects were not interfering with the translation of assayed proteins of other functions. However, EM images of MCP1_TP901-1_::Ter showed DNA-packaged capsids only with no tails attached, suggesting that virion assembly or stability is affected by the absence of MCP1_TP901-1_ (Fig 3B). Attempts to assign a function to MCP1_TP901-1_ through bioinformatic analysis highlighted that the N-terminal portion of MCP1_TP901-1_ and the complete MCP1_Tuc2009_ protein share a very high level (94.3%) of aa similarity. However, MCP1_TP901-1_ possesses an approximate 28 kDa C-terminal extension, or an additional 248 aa, which is lacking in MCP1_Tuc2009_. The initial ~100 aa of this 248 aa extension of MCP1_TP901-1_ appears to be due to a genetic duplication of a 5’-part of the MCP1_TP901-1_-encoding gene (data not shown). The remaining C-terminally located 145 amino acids of MCP1_TP901-1_ are lacking in its equivalent in Tuc2009, and no significant function can be ascribed to this C-terminal region based on database searches. The genes specifying MCP1_TP901-1_ and MCP1_Tuc2009_, as well as SPP1 capsid protein gp7 (gp7_SPP1_) all occur immediately downstream of the gene encoding their corresponding portal protein, and have a strong positive charge (pI values of ~9.0), and are largely α-helical (data not shown). Furthermore, ClustalW alignment of MCP1_TP901-1_, MCP1_Tuc2009_ and gp7_SPP1_ highlights sequence conservation, with the exception of the C-terminal extension of MCP1_TP901-1_ (S1 Fig).

Intact virions were observed during EM analysis of mutant MCP2_TP901-1_::Ter (Fig 3C); this was expected as the MCP2_TP901-1_::Ter mutant phage was shown to be still capable of forming plaques and establishing lysogeny of host *L*. *lactis* strain 3107 (Fig 1 and Table 2, respectively). ORF35 of TP901-1 (designated here as Sfp_TP901-1_) is annotated in this study as specifying a putative scaffolding protein (Sfp) based on the localization within the capsid-encoding structural module region and the predicted secondary structure of its corresponding protein product. Scaffolding proteins are involved in assisting the correct assembly of viral capsid structures by ensuring specific protein-protein interactions, nucleating the capsid assembly within cells, and preventing/excluding non-specific interactions between proteins during the molecular construction of phage capsids [35]. Sfp_TP901-1_ is predominantly α-helical and part of its structure is predicted to be intrinsically disordered (S2 Fig), features commonly associated with phage scaffolding proteins [35]. The translation terminating mutation in Sfp_TP901-1_ was shown to result in a decreased amount of the upstream-situated Portal_TP901-1_ protein_,_ and very little MHP_TP901-1_, detected in Western blots (Fig 2). This supports the prediction that Sfp_TP901-1_ acts as a scaffolding protein, interacting with essential capsid components to coordinate correct assembly.

Immunoblot examination of mutant MHP_TP901-1_ also revealed a decrease in the level of detected Portal_TP901-1_ and a complete absence of Sfp_TP901-1_, both of whose corresponding genes are situated upstream. The absence/reduction in capsid-related proteins in the PEG-precipitates of these mutants suggests that the proteins are produced, but are not assembled into the appropriate complexes. Consistent with this notion it was shown that tail structures, but no capsids, are present in Western blot and EM images of mutants Sfp_TP901-1_::Ter and MHP_TP901-1_::Ter (Figs 2, 3D and 3E, respectively).

Popovic *et al*. (2012) identified sequence identity between MCP3_TP901-1_ and phage λ capsid chaperone protein gpFI, which facilitates the interaction of DNA-terminase holoenzyme complexes with procapsids [86]. While Popovic and colleagues proposed MCP3_TP901-1_ is associated with phage capsids through a -1 ribosomal frameshift at the 3’ end of MHP_TP901-1_, a feature observed for several *Listeria*-infecting phages [86, 87], Western blots against MHP_TP901-1_ did not reveal a second band. HHpred analysis of MCP3 of TP901-1, and the analogous MCP3 of Tuc2009, revealed homology to GP10 of a T7-like *Podoviridae* phage infecting *Prochlorococcus* (e-value of 1.5e^-12^), and also to the major capsid protein gp5 of the well characterized phage HK97 (e-value of 5.8e^-05^). MCP3_Tuc2009_ exhibits similarity to a 52 aa region located at the C-terminus of HK97 gp5, corresponding to residues 332–384 of this viral procapsid protein. Amino acid Asn356 of gp5, conserved in MCP3_Tuc2009_, has been shown to be involved in covalently bonding neighbouring HK97 capsid subunits via an isopeptide bond [88]. Covalent cross-linkage of capsid protein subunits has previously been observed for lactococcal phage r1t [89], though it has not been documented to date for phages TP901-1 or Tuc2009. Consistent with the literature and bioinformatic analyses, MCP3_TP901-1_ is a non-essential protein that aids in efficient phage infectivity. This conclusion is supported by the results demonstrating mutants of this gene produce infective phages with a lower EOP that are still capable of establishing lysogeny (Fig 1 and Table 2, respectively), and full phages were observed in EM images (Fig 3F).

In addition to HTC1 being absent in mutant HTC1_TP901-1_::Ter, as expected, this protein is also absent in PEG-precipitated lysates of mutants Portal_TP901-1_::Ter, Sfp_TP901-1_::Ter and MHP_TP901-1_::Ter. EM images of HTC1_TP901-1_::Ter produced loose capsids and tails (Fig 3G). Analysis of the CsCl gradient-purified band of mutant HTC2_TP901-1_::Ter revealed, similar to what was observed for HTC1_TP901-1_::Ter, unconnected capsids and tails (Fig 3H), confirming the role of these proteins being required for head-tail connection.

Although no significant similarities to other proteins were detected by means of database searches, ORF40 of TP901-1 is annotated in this study as a Tail-activator protein (Tap_TP901-1_; see Discussion). DNA-packaged capsids and tail structures were visualized in the EM analysis of CsCl gradient-purified mutant Tap_TP901-1_::Ter, however, these structures were not connected (Fig 3I). Interestingly, EM images of the mutant Ttp_TP901-1_::Ter (ORF41), with a stop codon introduced into the gene putatively encoding the Tail terminator protein (Ttp), showed DNA-packaged capsids with long polytail structures (Fig 3J), possibly due to uncontrolled tail-tube polymerization, consistent with the absence of a Ttp.

Analysing the TP901-1*erm* mutants for the production of MTP showed that lysates of all presumed capsid mutants contained tail proteins, a clear indication that these mutations do not cause a negative polar effect on the RNA stability or translation of genes that were located downstream of such mutations (Fig 2). No MTP was detected in mutants of MTP_TP901-1_, gpG_TP901-1_ and gpT_TP901-1_. However, MTP was detected in the TP901-1 mutant permanently fusing gpG and gpT production together (gpG*fs*T_TP901-1_). EM images of mutant MTP_TP901-1_::Ter resulted, as expected, in tail-less capsids (Fig 3K).

In addition to detecting MTP_TP901-1_, Western blots were performed to detect the distal tail protein Dit_TP901-1_ in mutant lysates, as it is known to be crucial for the formation of functional phage tails [9]. Interestingly, Dit_TP901-1_ was undetectable in mutants MTP_TP901-1_, gpG_TP901-1_ and gpT_TP901-1_, where MTP_TP901-1_ was also absent (Fig 2), indicating that tail assembly was affected in these mutants.

The TAC proteins gpG and gpT of TP901-1, are expected to fulfil a similar role as phage λ gpG and gpT TAC proteins based on genetic synteny with lambdoid-like phages and the presence of a conserved ‘slippery’ sequence that is associated with gpGT production [49, 50]. Furthermore, a recent study focusing on the structural conservation and identification of TACs supported the annotation of gpG_TP901-1_ since the conserved spiral structure that typifies these TACs was maintained in the TP901-1 gpG equivalent [90]. EM visualization of the TP901-1*erm* carrying the gpG_TP901-1_::Ter revealed DNA-containing capsids and large disordered aggregates (Fig 3L). We propose that these aggregates, not present in any of the other analyzed phage preparations, are hydrophobic TMP molecules incapable of remaining in solution in the absence of the chaperone protein gpG. Analysis of mutant gpT_TP901-1_::BamHI revealed only the presence of phage capsids (Fig 3M). Interestingly, apparently complete phage particles were observed for mutant gpG*fs*T_TP901-1_ (Fig 3N). Despite having the appearance of wild-type TP901-1*erm*, mutant gpG*fs*T_TP901-1_ is incapable of infection (as determined by its inability to form plaques and lysogens) indicating aberrant tail assembly. Understanding the role(s) of gpG and gpGT may require higher resolution images of this tail assembly mutant and a better understanding of the chaperone-TMP interaction.

## Discussion

Members of the *Siphoviridae* family represent a distinct morphological group of tailed phages which are extremely efficient at infecting prokaryotes. In this study, we present data on the structural components of a *Siphoviridae* virus, TP901-1. Roughly half of the genome of TP901-1 is dedicated to the production of its structural components and their efficient assembly into an infectious virion. *In vitro* assembly of TP901-1*erm* structural module mutants, assessed by monitoring the frequency of lysogeny, delineated the boundaries between the TP901-1 genetic modules associated with capsid and tail morphogenesis. The organization of the TP901-1 genome co-locates all essential capsid protein-encoding genes at the beginning of the phage structural module, while genes required for tail production begin at *tap*, the gene specifying the putative Tail-activator protein, and continue as far as the host-specificity determining receptor binding protein-encoding gene *bppL* (Fig 1). This function-specific organization is fully consistent with the modular theory of phage genome organization [91].

Previous studies of phage λ have demonstrated that capsids and tails assemble independently, and that they can be joined together *in vitro* to form functional phages [85]. *In vitro* assembly of TP901-1*erm* similarly demonstrated that an MHP mutant produces functional tail structures, while the MTP mutant still forms intact DNA-packaged capsids. No additional host factors appear to be required for the connection of phage capsids and tails, as these individual multiprotein complexes appear to be ready to assemble once formed. The lysogeny frequency of *in vitro* assembled virions generated by mixing purified mutants MHP_TP901-1_::Ter and MTP_TP901-1_::Ter, relative to that of a TP901-1*erm* phage control, tells us that the efficiency of assembly under the tested conditions is approximately 2.5–3.0% (Table 2). The assembly of TP901-1*erm* capsids and tails is thus remarkably efficient, assuming that the MHP mutant (donating phage tails) and the MTP mutant (donating phage capsids) behave similarly to complete TP901-1*erm* virions during purification, and contribute the necessary structures at an optimal ratio. Therefore, by optimizing the *in vitro* assembly parameters, the achievable efficiency of phage capsid and tail assembly may perhaps be even higher.

**Fig 4:**
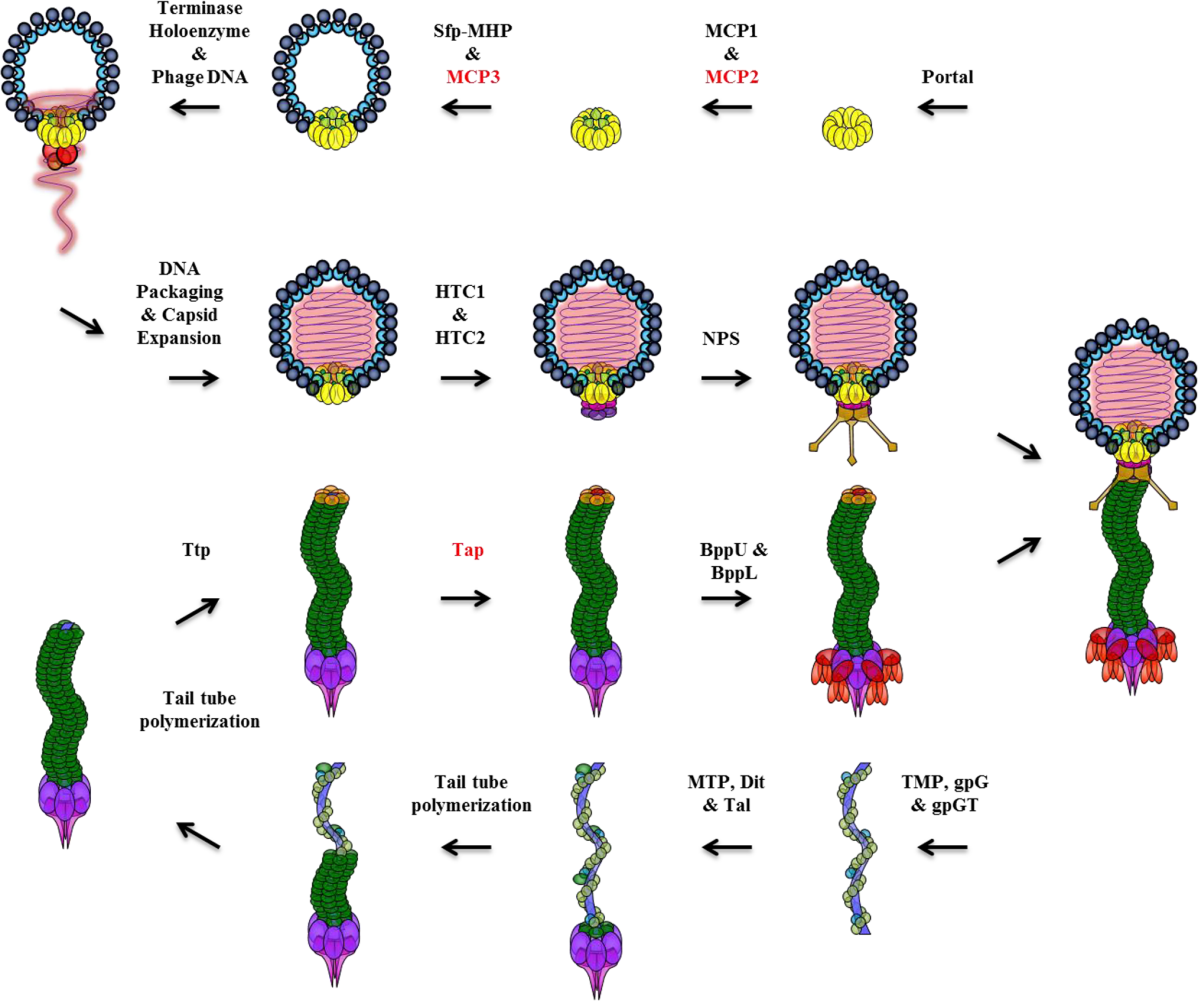
Molecular model depicting TP901-1 phage assembly. See Discussion section for details. Protein names in red are putatively depicted as virion-comprising proteins; however, their localization and copy number, if indeed present, is currently unknown. Colours assigned to proteins correspond to Fig 1.

Western blot detection of TP901-1 proteins demonstrated the strong functional relationships between virion-composing proteins, how these interactions are both complex and essential, and facilitated a model for the assembly of TP901-1 virion (Fig 4). In this model, the portal protein of TP901-1 is important as it is predicted to initiate capsid assembly through interactions with other crucial capsid components. MCP1_TP901-1_ is presumed to act as a functional analogue of phage SPP1 capsid protein gp7 (see Results section) and thus is predicted to associate with the internal face of the portal protein complex. SPP1 gp7, present at an estimated three copies per virion, is proposed to bind DNA in a non-specific manner to control genome ejection at the onset of infection [92–94]. The *in vitro* assembly and lysogeny assays of TP901-1 mutants suggests that mutant MCP1_TP901-1_ assembles into full virions, as the mutation could not be efficiently complemented by the addition of purified capsids or tails. However, achieving high-quality EM images of MCP1_TP901-1_::Ter to confirm this result was not possible, as ultracentrifugation preparations of this mutant generated only capsids which appear devoid of DNA (see Fig 3B). Therefore, more work is required to understand the role of MCP1_TP901-1_ in viral assembly and possibly its role in virion stability. MCP2_TP901-1_ is depicted as a capsid protein in our phage assembly model; however, also in this case further work is required to confirm that MCP2 is indeed part of mature TP901-1 virions.

Analysis of presence and abundance of Portal_TP901-1_, Sfp_TP901-1_ and MHP_TP901-1_ in various TP901-1*erm* mutants indicates that these proteins orchestrate the correct assembly and/or stabilization of capsid structures (Fig 2). There is a pronounced lack of the Sfp_TP901-1_ in mutant MHP_TP901-1_::Ter, and *vice versa*, suggesting that these two proteins are closely connected during TP901-1 capsid assembly (Fig 4). DNA packaging within procapsids of TP901-1 by the terminase holoenzyme, or stabilization of the formed capsid structures, may be facilitated by MCP3. This is due to MCP3 sharing homology to known capsid-assembly chaperone proteins and regions of characterized phage capsid proteins involved in covalent cross-linkage of subunits or potential interaction of MCP3 with the MHP as suggested by Popovic and colleagues (87) in the case of the MCP3 equivalent, gpFI of phage lambda. DNA packaged within TP901-1 procapsids likely expands the capsids and is a prerequisite for creating an infectious phage.

HTC1, and subsequently HTC2, are predicted to associate with TP901-1 capsids only once DNA is packaged. HTC1_TP901-1_, being absent in mutants Portal_TP901-1_::Ter, Sfp_TP901-1_::Ter and MHP_TP901-1_::Ter, appears to be a functional analogue of SPP1 gp15, and can fit into the electron density map of the TP901-1 head-tail connector region during the pseudoatomic model construction of this phage [14, 42]. Interestingly, SPP1 gp15 only associates with its cognate portal protein once DNA packaging is complete [42]; therefore, the detection of HTC1_TP901-1_ in mutant phages MCP1_TP901-1_::Ter, MCP2_TP901-1_::Ter and MCP3_TP901-1_::Ter suggests that these mutants all produce DNA packaged capsids. Our EM analyses of MCP1_TP901-1_::Ter, showing DNA packaged capsids (Fig 3B), and MCP2_TP901-1_::Ter and MCP3_TP901-1_::Ter, displaying complete phages with DNA-packaged capsids (Fig 3C and 3F, respectively), are in agreement with this notion. Capsids of TP901-1, ready to assemble to their cognate tail structures, contain non-essential fibres or neck-passage structures (NPS) associated with the portal vertex (Fig 4; [10, 56]).

The assembly of the TP901-1 tail organelle is depicted in our phage assembly model as beginning with the TMP and chaperones gpG and gpGT (Fig 4). Recent structural studies and biochemical investigations of tail chaperone proteins have provided information on the roles of gpG and gpGT (Fig 4; [48, 49, 90, 95]). Preventing translation of TP901-1*erm* gpG or gpT (mutants gpG_TP901-1_::Ter and gpT_TP901-1_::BamHI, respectively) results in a failure to detect MTP or baseplate protein Dit. This is in agreement with the results obtained by Xu *et al*. (2013) who demonstrated that gpG of phage λ is bound to the TMP, while the ‘T’ domain of gpGT recruits the phage MTP to the assembling tail complex [49]. Interestingly, analysis of a mutation that mimics the natural frame-shift, yet permanently fusing genes *gpG* and *gpT* to produce protein gpGT (mutant gpG*fs*T_TP901-1_; S2 Table) showed that all tail proteins tested by Western blotting are present and that morphologically intact phage particles seem to be produced; however, these particles did not exhibit any infectivity. This confirms previous observations whereby production of a specific ratio of gpG to gpGT is essential in order to form functional tail structures [49, 50], possibly by facilitating MTP and/or TMP to adopt a conformation that allows signal transfer to the portal protein following host recognition.

While the interactions of tail proteins during assembly are likely rapid or occur simultaneously, the TP901-1 initiator complex, composed of TMP, Dit and the N-terminus of Tal protein, is proposed as a prerequisite to tail tube formation (Fig 4; [9]). Structural studies of phage λ gpV showed that the tail tube-forming protein remains in a monomeric form in solution at high concentrations, but gpV is thought to polymerize upon contact with the tail initiator complex as it provides a scaffold for polymerization and ordering of unstructured regions [82]. Thus, polymerization of TP901-1 MTP likely initiates at the tail tip and proceeds towards the capsid-proximal end of the phage tail.

As stated above, no function could be ascribed to the protein Tap_TP901-1_ based on database searches. The nomenclature and proposed function of Tap_TP901-1_ was based purely on its observed role in joining capsids and tails (Fig 3H and Table 2). However, it is not yet known whether Tap_TP901-1_ is part of the mature TP901-1 virion structure. Based on EM analysis, Tap_TP901-1_ appears to play a similar role to gpZ_λ_ in tail morphogenesis, as mutations in the encoding genes produce seemingly intact tails and capsids, which fail in joining together [51].While depicted as the final step in the production of TP901-1 tails, proteins BppU and BppL are likely to associate with Dit immediately after the initiation complex forms to create the baseplate structure which functions as the phage host-recognising receptor-binding complex.

In conclusion, our current and previous findings highlight conserved mechanisms of structural protein assembly and functionality between the lactococcal phages TP901-1 and Tuc2009 on the one hand, and other well characterized *Siphoviridae* phages, consistent with the notion that such phages have evolved from a common origin of efficient DNA-delivery machines.

## Supporting Information

S1 Fig ClustalW alignment of phage capsid proteins MCP1_TP901-1_, MCP1_Tuc2009_ and gp7_SPP1_. The three capsid proteins share significant conservation, except for the approximate 28 kDa C-terminal extension present in MCP_TP901-1_.
(PDF)

S2 Fig Secondary structure and intrinsically disordered-region predictions of (A) TP901-1, (B) Tuc2009, (C) phage λ and (D) phage SPP1 scaffolding, or scaffolding-like, proteins. Sfp_TP901-1_, Sfp_Tuc2009_, gpNu3_λ_ and gp11_SPP1_ are all predicted as largely α-helical in structure (PSIPRED) and contain large regions of intrinsically disordered protein structure (IUPRED).
(PDF)

S1 Table Bacterial strains, phages and plasmids used in this study. The names, relevant features and source (if applicable) of all bacterial strains, phages and plasmids used in this study.
(PDF)

S2 Table Detailed description of the mutations created in the various strains during this study, and their effects. NCBI accession numbers and the genomic coordinates of regions targeted for mutation are listed. The original and following-mutagenesis sequences of TP901-1*erm* structural module genes and proteins are highlighted.
(PDF)

S3 Table Oligonucleotides used in this study. Sequences of oligonucleotides used to: create mutations via recombineering, PCR screen for the various mutations and generate recombinant protein constructs.
(PDF)
