# Supplementary material for: Structure and Assembly of TP901-1 Virion Unveiled by Mutagenesis
Source: PLoS One. 2015 Jul 6;10(7):e0131676. doi: 10.1371/journal.pone.0131676 (PMC4493119; doi:10.1371/journal.pone.0131676)
Supplement: S1 Fig — The three capsid proteins share significant conservation, except for the approximate 28 kDa C-terminal extension present in MCPTP901-1. (PDF) [file pone.0131676.s001.pdf]

```
MCP1-TP901-1      MKTPDYWKKREKAWQEQQIKDDTKRMKQIMDKLFEAQEAIQKEINANWQNFANGQGISIS
MCP1-Tuc2009      MNSSDYWRKREKAWQEQQIKDDTKRMKQIMDKLFEAQEAIQKEINANWQNFANGQGISIS
gp7-SPP1          MPEPQNQEELDKYLDN-IITQAEKRLDKVFASRLKEIKAMINKLFEKYS--KNGE-----
                  *  .:  .: :*  ::  *.:  **:.::: . ::  :*: :::  ::.   **:

MCP1-TP901-1      EAMKRADKMDVKAFANKAKKYVQEKDFSNQANQVLKLYNLTMRVNRLELLKANIGLELIS
MCP1-Tuc2009      EAMKRADKMDVKAFENKAKKYVKEKDFSHQANQVLKLYNLTMRVNRLELLKANIGLELIS
gp7-SPP1          --LTYADVVKYNRLEKEMD--VIKQNISADYKTVLKMLNELLETQYVDNYLR--SAYIYE
                    :. ** :. : : :: .  * ::::* : : ***: *  :..: ::      .  : .

MCP1-TP901-1      VFDDLDKYFSNNLTSAALTEFERQAGILGLSVPKEGYNSLVESVLNGSYKVEGFASFSDK
MCP1-Tuc2009      VFDDLDKYFSKNLTGAALTEFERQAGILGLSVPKKGYNSLVESVLNGSYKVEGFASFSDK
gp7-SPP1          MYTGRNLGFS--VPSADVVQRAVENPIPLLTLPKVLERQRVELINNIAMAIAQGLMAGEG
                  :: . :  **  :..* :.:   :  *  *::**   .. ** : * :  :      .:

MCP1-TP901-1      LWQYQFELKADIEKLLIRSVTGGINPKTLAPQLKRLMTEKGKLNATYNAQRLLVSETTRI
MCP1-Tuc2009      IWQYQFELKADIEKLLIRSVTGGINLKALAPQLKRLMTEKGKLNATYNAQRLLVSETTRI
gp7-SPP1          YSQVAQRVHKRMQLSLAKARLTARTEGHRVQVAGRMASAEQAAKKVNMQKMWSAALDTRT
                    *   .::  ::  * ::   . .    .    *: : :   : .   :   .:  **

MCP1-TP901-1      QTAIQEESYKKADIESYEYIAEPSACPICGALNGKIFKLKDMSPGINAPNMHPFCRCSTA
MCP1-Tuc2009      QTAIQEESYKKADIESYEYIAEPSACPICGALNGKIFKLKDMSPGINAPNMHPFCRCSTA
gp7-SPP1          RAGHRKLDGKIINMD-------ENFKSIYGGVGKAPGHMHMAKDDCNCRCALIYVIDGEI
                  ::. :: . *  :::        .  .* *.:.    :::  . . *.     :   .

MCP1-TP901-1      PHVDDKAFWDDRLKEQDKKNSGKNIPVSLKGLNDDYLNEKREESRLKAGRVNAKKYDAQS
MCP1-Tuc2009      PHVDDKGFWDDLLD----------------------------------------------
gp7-SPP1          PSVRRARLSDGSTR----------------------------------------------
                  * *    : *.

MCP1-TP901-1      ESFANVTNEASMRMRISDIGFRRAIESGNLKSSHELGDDFDKGRIRIEKTLFNLPENIKR
MCP1-Tuc2009      ---------------------RKVISQDEYKQA---------------------------
gp7-SPP1          --------------------VIKYIPYTEWEKQ---------------------------
                                        : *   : :.

MCP1-TP901-1      SEMPKYGYLSDSDDLFEKKTKHSVLGYGNIIIELDDSVRKRTTYTVNDSLVNKRGLITSA
MCP1-Tuc2009      ---------------FDDRTED--------------------------------------
gp7-SPP1          -----------------KKAS---------------------------------------
                                   .::.

MCP1-TP901-1      TPVGTKPTYNGIKEKAIGEINSISEFLNSNKKTNRYIEAQYHGDLTFKNDVKRIIVPDKS
MCP1-Tuc2009      -------------DKAIEELR--------NKRKG--------------------------
gp7-SPP1          ------------------------------------------------------------

MCP1-TP901-1      YLDKLSKEFEQLKNMGIEVLVAPK
MCP1-Tuc2009      ------------------------
gp7-SPP1          ------------------------
```
