# Supplementary material for: Structure and Assembly of TP901-1 Virion Unveiled by Mutagenesis
Source: PLoS One. 2015 Jul 6;10(7):e0131676. doi: 10.1371/journal.pone.0131676 (PMC4493119; doi:10.1371/journal.pone.0131676)
Supplement: S2 Fig — SfpTP901-1, SfpTuc2009, gpNu3λ and gp11SPP1 are all predicted as largely α-helical in structure (PSIPRED) and contain large regions of intrinsically disordered protein structure (IUPRED). (PDF) [file pone.0131676.s002.pdf]

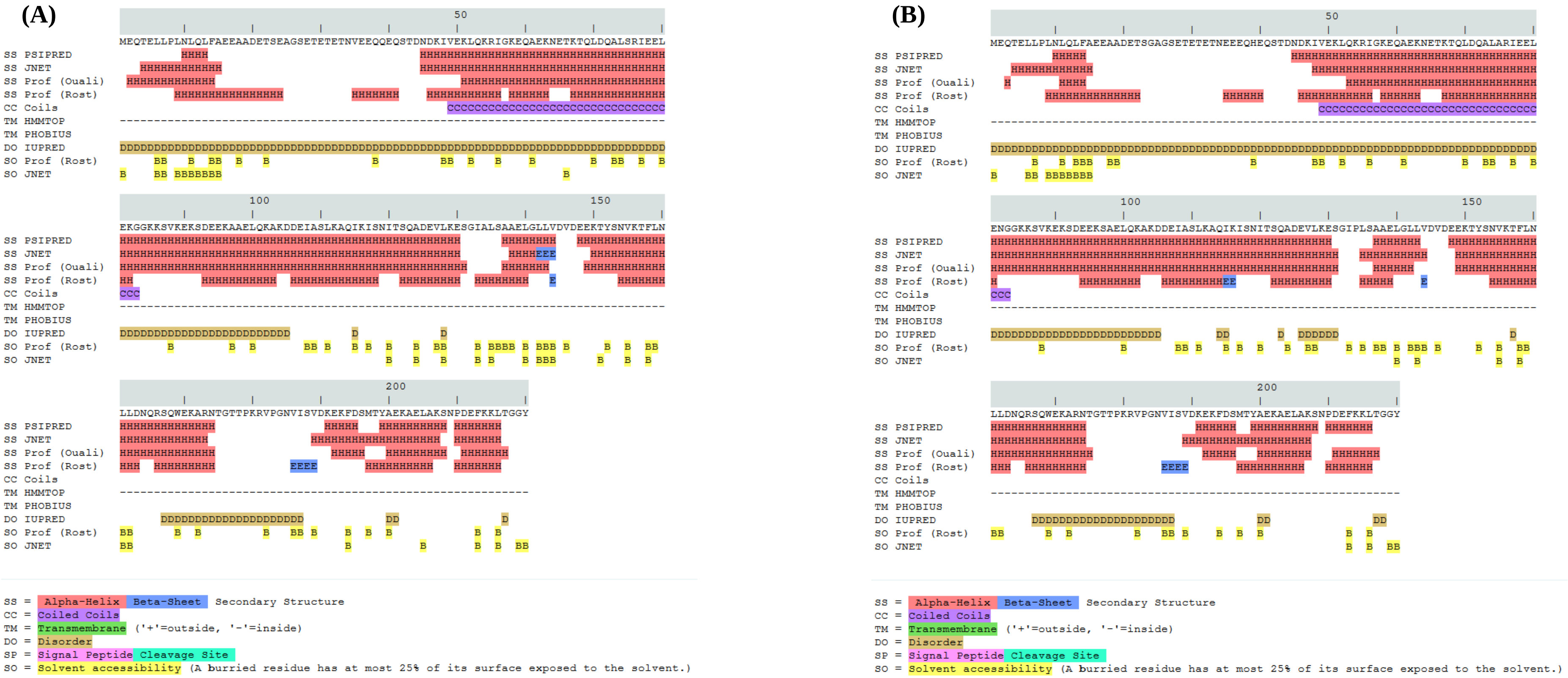

(A)
(B)
SS PSIPRED
SS JNET
SS Prof (Ouali)
SS Prof (Rost)
CC Coils
TM HMMTOP
TM PHOBIUS
DO IUPRED
SO Prof (Rost)
SO JNET
SS = Alpha-Helix Beta-Sheet Secondary Structure
CC = Coiled Coils
TM = Transmembrane ('+'=outside, '-'=inside)
DO = Disorder
SP = Signal Peptide Cleavage Site
SO = Solvent accessibility (A burried residue has at most 25% of its surface exposed to the solvent.)

(C)

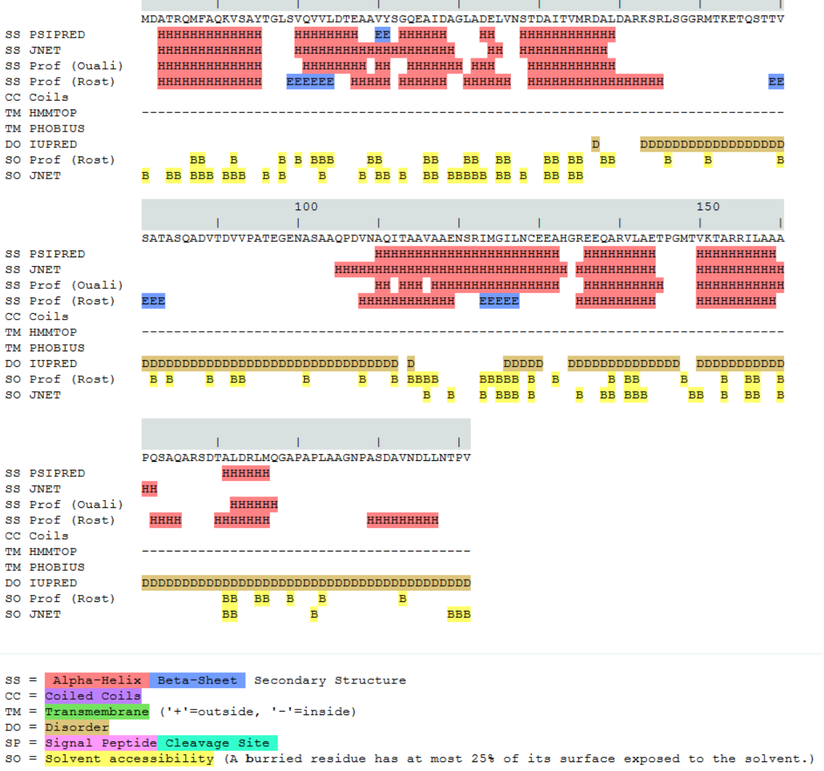
50
MDATRQMFAQKVSAYTGLSVQVVLDTEAAVYSGQEAIDAGLADELVNSTDAITVMRDALDARKSRLSGGRMTKETQSTTV
100
150
SATASQADVTDVVPATEGENASAAQPDVNAQITAAVAAENSRIMGILNCEEAHGREEQARVLAETPGMTVKTARRILAAA
PQSAQARSDTALDRLMQGAPAPLAAGNPASDAVNDLLNTPV
SS PSIPRED
SS JNET
SS Prof (Ouali)
SS Prof (Rost)
CC Coils
TM HMMTOP
TM PHOBIUS
DO IUPRED
SO Prof (Rost)
SO JNET
SS = Alpha-Helix Beta-Sheet Secondary Structure
CC = Coiled Coils
TM = Transmembrane ('+'=outside, '-'=inside)
DO = Disorder
SP = Signal Peptide Cleavage Site
SO = Solvent accessibility (A burried residue has at most 25% of its surface exposed to the solvent.)

(D)

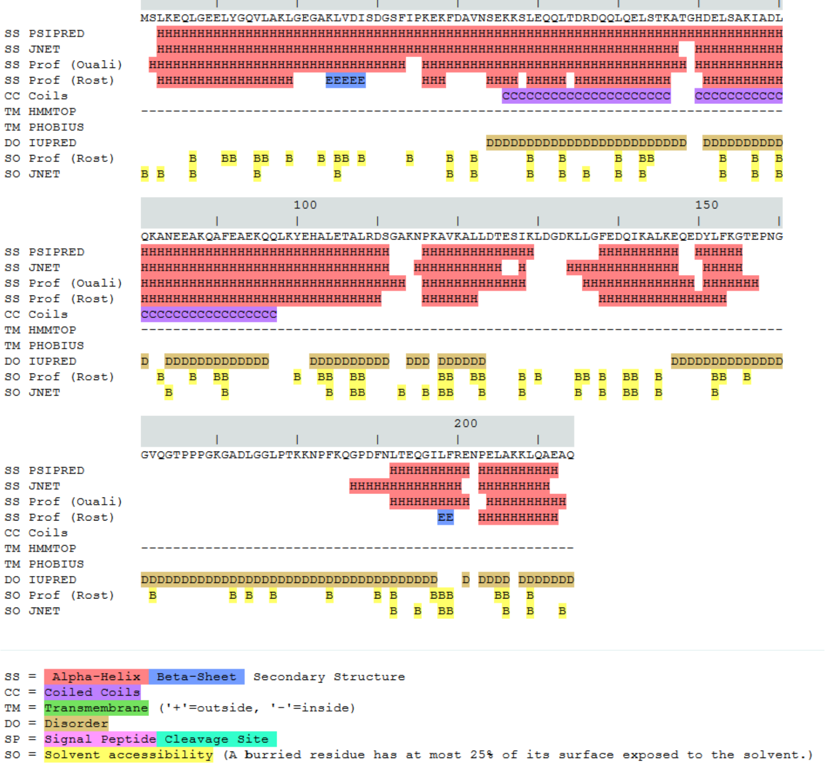
50
MSLKEQLGEELYGQVLAKLGEGAKLVDISDGSFIPKEKFDAVNSEKKSLEQQLTDRDQQLQELSTKATGHDELSAKIADL
100
150
QKANEEAKQAFEAEKQQLKYEHALETALRDSGAKNPKAVKALLDTESIKLDGDKLLGFEDQIKALKEQEDYLFKGTEPNG
200
GVQGTPPPGKGADLGGLPTKKNPFKQGPDFNLTEQGILFRENPELAKKLQAEAQ
SS PSIPRED
SS JNET
SS Prof (Ouali)
SS Prof (Rost)
CC Coils
TM HMMTOP
TM PHOBIUS
DO IUPRED
SO Prof (Rost)
SO JNET
SS = Alpha-Helix Beta-Sheet Secondary Structure
CC = Coiled Coils
TM = Transmembrane ('+'=outside, '-'=inside)
DO = Disorder
SP = Signal Peptide Cleavage Site
SO = Solvent accessibility (A burried residue has at most 25% of its surface exposed to the solvent.)
