# Supplementary material for: Structure and Assembly of TP901-1 Virion Unveiled by Mutagenesis
Source: PLoS One. 2015 Jul 6;10(7):e0131676. doi: 10.1371/journal.pone.0131676 (PMC4493119; doi:10.1371/journal.pone.0131676)
Supplement: S1 Table — The names, relevant features and source (if applicable) of all bacterial strains, phages and plasmids used in this study. (PDF) [file pone.0131676.s003.pdf]

Strains, phages and plasmids	Relevant features	Source
L. lactis		
NZ9000	MG1363 *pepN::nisRK*	(51)
NZ9000-Cro$_{t712}$	NZ9000 with point mutations inserting a BamHI site into prophage t712 anti-repressor (Cro) promotor sequence; contains plasmid pJP005	This study
NZ9000-Cro$_{t712}$-TP901-1*erm*	NZ9000-Cro$_{t712}$ displaying t712^{-} phenotype lysogenized with TP901-1*erm* prophage; contains plasmid pJP005	This study
901-1	Lysogenic host and source of TP901-1 prophage	(13)
3107	Lytic host for phage TP901-1*erm*	(23)
UC509	Lysogenic host and source of Tuc2009 prophage	(5)
UC509.9	Lytic host for phage Tuc2009	(1, 25)
NZ9700	Nisin over-producing strain	(50)
NZ9000-Cro$_{t712}$-Portal$_{TP901-1}$::Ter	Translation termination codon insertion in TP901-1*erm* portal protein (ORF32)	This study
NZ9000-Cro$_{t712}$-MCP1$_{TP901-1}$::Ter	Translation termination codon insertion in TP901-1*erm* MCP1 (ORF33)	This study
NZ9000-Cro$_{t712}$-MCP2$_{TP901-1}$::Ter	Translation termination codon insertion in TP901-1*erm* MCP2 (ORF34)	This study
NZ9000-Cro$_{t712}$-Sfp$_{TP901-1}$::Ter	Translation termination codon insertion in TP901-1*erm* Sfp (ORF35)	This study
NZ9000-Cro$_{t712}$-MHP$_{TP901-1}$::Ter	Translation termination codon insertion in TP901-1*erm* MHP (ORF36)	This study
NZ9000-Cro$_{t712}$-MCP3$_{TP901-1}$::Ter	Translation termination codon insertion in TP901-1*erm* MCP3 (ORF37)	This study
NZ9000-Cro$_{t712}$-HTC1$_{TP901-1}$::Ter	Translation termination codon insertion in TP901-1*erm* HTC1 (ORF38)	This study
NZ9000-Cro$_{t712}$-HTC2$_{TP901-1}$::Ter	Translation termination codon insertion in TP901-1*erm* HTC2 (ORF39)	This study
NZ9000-Cro$_{t712}$-Tap$_{TP901-1}$::Ter	Translation termination codon insertion in TP901-1*erm* Tap (ORF40)	This study
NZ9000-Cro$_{t712}$-Ttp$_{TP901-1}$::Ter	Translation termination codon insertion in TP901-1*erm* Ttp (ORF41)	This study
NZ9000-Cro$_{t712}$-MTP$_{TP901-1}$::Ter	Translation termination codon insertion in TP901-1*erm* MTP (ORF42)	This study
NZ9000-Cro$_{t712}$-gpG$_{TP901-1}$::Ter	Translation termination codon insertion in TP901-1*erm* gpG (ORF43)	This study
NZ9000-Cro$_{t712}$-gpT$_{TP901-1}$::BamHI	Insertion mutation in TP901-1*erm* gpG (ORF43) disrupting gpT (ORF44) production	This study
NZ9000-Cro$_{t712}$-gpG*fs*T$_{TP901-1}$	Insertion and nucleotide altering mutation in TP901-1*erm* gpG (ORF43) causing a frameshift and permanently fusing it in frame to gpT (ORF44)	This study
**Phages**		
TP901-1*erm*	Temperate P335 species phage infecting 3107; contains erythromycin marker	(13, 48, 94)
Tuc2009	Temperate P335 species phage infecting UC509.9	(25)
**Plasmids**		
pNZ8048	Nisin-inducible protein expression vector	(27)
pTX8049	pNZ8048 derivative expression vector; recombinant N-terminal thioredoxin fusion	(31)
pJP005	pNZ8048 derivative expressing RecT protein required for recombineering mutagenesis	(86)
pNZ8048-Portal$_{Tuc2009}$	Tuc2009 Portal protein (ORF33) expression construct; Genbank coordinates 16486-17847	This study
pNZ8048-MCP1$_{Tuc2009}$	Tuc2009 MCP1 (ORF34) expression construct; Genbank coordinates 17844..18884	This study
pTX8049-MCP2$_{Tuc2009}$	Tuc2009 MCP2 (ORF35) expression construct; Genbank coordinates 18930..19262	This study
pNZ8048-Sfp$_{Tuc2009}$	Tuc2009 Sfp (ORF36) expression construct; Genbank coordinates 19388..20050	This study
pTX8049-MHP$_{Tuc2009}$	Tuc2009 MHP (ORF37-39) expression construct; Genbank coordinates 20052..22183	This study
pTX8049-MCP3$_{Tuc2009}$	Tuc2009 MCP3 (ORF40) expression construct; Genbank coordinates 22183..22383	This study
pTX8049-HTC1$_{Tuc2009}$	Tuc2009 HTC1 (ORF41) expression construct; Genbank coordinates 22367..22699	This study
pTX8049-Tap$_{Tuc2009}$	Tuc2009 Tap (ORF43) expression construct; Genbank coordinates 23004..23330	This study
