# Supplementary material for: Structure and Assembly of TP901-1 Virion Unveiled by Mutagenesis
Source: PLoS One. 2015 Jul 6;10(7):e0131676. doi: 10.1371/journal.pone.0131676 (PMC4493119; doi:10.1371/journal.pone.0131676)
Supplement: S2 Table — NCBI accession numbers and the genomic coordinates of regions targeted for mutation are listed. The original and following-mutagenesis sequences of TP901-1erm structural module genes and proteins are highlighted. (PDF) [file pone.0131676.s004.pdf]

Mutant strain and phages	Accession numbers, ORF numbers and genomic coordinates of mutation region	Original DNA sequence	Original protein sequence[1, 2]	DNA sequence following recombineering[3]	Protein sequence following recombineering
L. lactis **NZ9000**	**NC_017949**				
NZ9000-Cro$_{t712}$	782805..782833	TTGACAAGTTGCAACGA CGATGTTATAAT	N/A	TTGACAAGTTGCAACGA CGATCAGGATCC	N/A
**TP901-1**	**NC_002747**				
NZ9000-Cro$_{t712}$-Portal$_{TP901-1}$::Ter	ORF32; 15816..15839	AAACAAGAGCCTTTATT TGCCGTG	KQEPLFAV	AAACAAGAGCCTTGAA TTCTATTTGCCGTG	KQEP*ILFAV
NZ9000-Cro$_{t712}$-MCP1$_{TP901-1}$::Ter	ORF33; 17151..17174	GGAATTCTTGGTTTAAG CGTTCCA	GILGLSVP	GGAATTCTTGGTTGAAT TCATAAGCGTTCCA	GILG*IHKRS
NZ9000-Cro$_{t712}$-MCP2$_{TP901-1}$::Ter	ORF34; 18500..18523	TATCATTACTATTTTGA CGGTGAT	YHYYFDGD	TATCATTACTATTGAAT TCATTGACGGTGAT	YHYY*IH*R*
NZ9000-Cro$_{t712}$-Sfp$_{TP901-1}$::Ter	ORF35; 19028..19051	GTTAAAGAAAAATCTG ACGAAGAA	VKEKSDEE	GTTAAAGAAAAATGAA TTCCTGACGAAGAA	VKEK*IPDEE
NZ9000-Cro$_{t712}$-MHP$_{TP901-1}$::Ter	ORF36; 19812..19835	GCTAAGACTACCTCTCA AACTGTT	AKTTSQTV	GCTAAGACTACCTGAAT TCGCTCAAACTGTT	AKTT*IRSNC
NZ9000-Cro$_{t712}$-MCP3$_{TP901-1}$::Ter	ORF37; 20318..20341	GATGAACTAACGACGCT TACCGTT	DELTTLTV	GATGAACTAACGTGAAT TCACGCTTACCGTT	DELT*IHAYR
NZ9000-Cro$_{t712}$-HTC1$_{TP901-1}$::Ter	ORF38; 20526..20549	CTTATTCTTGGTTCTGA CATTAAA	LILGSDIK	CTTATTCTTGGTTGAAT TCCTGACATTAAA	LILG*IPDIK
NZ9000-Cro$_{t712}$-HTC2$_{TP901-1}$::Ter	ORF39; 20900..20923	AGTGTAGAAGTTTTTGG AGATATT	SVEVFGDI	AGTGTAGAAGTTTAGAA TTCTTGGAGATATT	SVEV*NSWRY
NZ9000-Cro$_{t712}$-Tap$_{TP901-1}$::Ter	ORF40; 21107..21130	CTTGTAAAGCATTTGGA TAAAGCA	LVKHLDKA	CTTGTAAAGCATTGAAT TCTGGATAAAGCA	LVKH*ILDKA
NZ9000-Cro$_{t712}$-Ttp$_{TP901-1}$::Ter	ORF41; 21444..21467	TTCAAACGAATCCAAGC TTTGGGG	FKRIQALG	TTCAAACGATGATGAGC TTTGGGG	FKR**ALG
NZ9000-Cro$_{t712}$-MTP$_{TP901-1}$::Ter	ORF42; 22061..22084	ATGCAGCAATTATTGAA GTGTGGG	MQQLLKCG	ATGCAGCAATTATGAAT TCATGAAGTGTGGG	MQQL*IHEVW
NZ9000-Cro$_{t712}$-gpG$_{TP901-1}$::Ter	ORF43; 22579..22602	AACATTGCAACTTTATC GAATGTA	NIATLSNV	AACATTGCAACTTGAAT TCTATCGAATGTA	NIAT*ILSNV
NZ9000-Cro$_{t712}$-gpT$_{TP901-1}$::BamHI	ORF44; 22731..22737	GAAAGCAATACGGGAA AGTTAATC	ESNTGKLI..[6 aa]..*	GAAAGCAATACGGGAT CCGGAAAGTTAATC	ESNTGSGKLI..[6 aa]..*
NZ9000-Cro$_{t712}$-gpG*fs*T$_{TP901-1}$	ORF43-44; 22731..22737	GAAAGCAATACGGGAA AGTTAATC	ESNTGKLI..[6 aa]..*	GAAAGCAATACTGGTA AAGCTGATC	ESNTGKVN..[126 aa]..*

1 The original protein sequence of NZ9000-Cro$_{t712}$-gpT$_{TP901-1}$::Ter mutant shows only the amino acid sequence of gpG. Protein sequence of gpGT (produced through a -1 frame-shift in *gpG* DNA sequence GGGAAAG, fusing it to *gpT*) is not shown.

2 The original protein sequence of NZ9000-Cro$_{t712}$-gpG*fs*T$_{TP901-1}$ mutant shows only the amino acid sequence of gpG.

3 Introduced DNA bases causing mutations are underlined once, while introduced bases resulting in a novel restriction site are double underlined.

N/A Not applicable

* Translation terminating stop codon
