# Supplementary material for: Structure and Assembly of TP901-1 Virion Unveiled by Mutagenesis
Source: PLoS One. 2015 Jul 6;10(7):e0131676. doi: 10.1371/journal.pone.0131676 (PMC4493119; doi:10.1371/journal.pone.0131676)
Supplement: S3 Table — Sequences of oligonucleotides used to: create mutations via recombineering, PCR screen for the various mutations and generate recombinant protein constructs. (PDF) [file pone.0131676.s005.pdf]

| Oligonucleotide name | Oligonucleotide sequence (5’ → 3’) |
|---|---|
| **Recombineering primers**[Ψ] | |
| NZ9000-Cro$_{t712}$::BamHI | C*T*C*C*A*TGATTGTTCGCTTGTTTGACTTTATGAGTTAGGATCCTGATCGTCGTTGCAACTTGTCAACCATTAAGGTTGTTTT |
| Portal$_{TP901\text{-}1}$::Ter | T*T*T*T*T*GTCCTCGTCAACACCATATCTCACGGCAAATAGAATTCAAGGCTCTTGTTTGACTGTATCGTCATAGACCATAAA |
| MCP1$_{TP901\text{-}1}$::Ter | T*C*A*A*C*TAAACTGTTATATCCTTCCTTTGGAACGCTTATGAATTCAACCAAGAATTCCGGCTTGTCTTTCAAATTCTGTGAG |
| MCP2$_{TP901\text{-}1}$::Ter | G*C*T*A*T*AATTTCTTCAACAACATTTCTATCACCGTCAATGAATTCAATAGTAATGATAGAATTTATTTTCTTTTTTAAAGATGA |
| Sfp$_{TP901\text{-}1}$::Ter | T*T*A*G*C*TTTTTGAAGTTCGGCAGCTTTTTCTTCGTCAGGAATTCATTTTTCTTTAACTGACTTTTTGCCACCTTTTTCAAG |
| MHP$_{TP901\text{-}1}$::Ter | C*C*C*C*G*TCAACGTTTGCTTTAGTAGAAACAGTTTGAGCGAATTCAGGTAGTCTTAGCTGCGCTCAATAAGTCGTCATCGA |
| MCP3$_{TP901\text{-}1}$::Ter | T*A*G*T*T*TCAAGAAGCTCTTTTAGCTGATTAACGGTAAGCGTGAATTCACGTTAGTTCATCATTTTTCACTTGCTTGGTCGCATTTATG |
| HTC1$_{TP901\text{-}1}$::Ter | A*C*A*T*A*TTCTAGTTCTGGCGGTACTTCTTTAATGTCAGGAATTCAACCAAGAATAAGCAATAAACGGTCACGAGTGCGTTT |
| HTC2$_{TP901\text{-}1}$::Ter | T*C*A*T*T*CGCATGACTTTTGCCCCTTTTTTAATATCTCCAAAGAATTCAAACTTCTACACTTCTGTCAGTTCCAATATCAGTGATATTT |
| Tap$_{TP901\text{-}1}$:: Ter | G*T*T*G*A*ACACCCTTTAAAGATGCTGCTTTATCCAGAATTCAATGCTTTACAAGTTGGTCAATCCCTTTAAAAGATAAGCTA |
| Ttp$_{TP901\text{-}1}$::Ter | T*C*A*T*T*GGCTTATAATCATAAACGGTATACCCCAAAGCTCATCATCGTTTGAACAATTCGTCAAAAATAGATTGGTCTCGAG |
| MTP$_{TP901\text{-}1}$::Ter | A*G*T*T*G*CTTTTTCTGCTTTATCAATTTCCCACACTTCATGAATTCATAATTGCTGCATCATCAAACGCTTTGTCCATTTCGT |
| gpG$_{TP901\text{-}1}$::Ter | G*T*T*T*C*TGTTCGATTTCCTAAAAATAATACATTCGATAGAATTCAAGTTGCAATGTTAGCCATTTCTAGTTCAGGAATGAT |
| gpT$_{TP901\text{-}1}$::BamHI | T*C*A*G*C*TATTTGGTCATTTTTGCTTTGATTAACTTTCCGGATCCCGTATTGCTTTCAGTAATTTCTTTCAAAACATCATCA |
| gpG*fs*T$_{TP901\text{-}1}$ | C*A*A*A*C*TTTTCAGCTATTTGGTCATTTTTGCTTTGATCAGCTTTACCAGTATTGCTTTCAGTAATTTCTTTCAAAACATCATCA |
| **Screening primers**[¥] | |
| Cro-t712-Fw | CTTCAAGATATTCATCGCTCAGACGG |
| Cro-t712-MidFw | TTGCAACGACGATCAGGATCC |
| Cro-t712-Rv | TCGTAATCATCAAGCGCATGTTCC |
| Scrnseq-Portal-Fw | GCAATGACAACTTATCAGCGTGA |
| Scrn-Portal -MidRv | CACCATATCTCACGGCAAATAGAATTC |
| Seq-Portal -Rv | ACTGGTAAATCTGGATATGGGTTG |
| Scrnseq-MCP1-Fw | GAGAAAGCTTGGCAAGAGCA |
| Scrn-MCP1-MidRv | CTTCCTTTGGAACGCTTATGAATTC |
| Seq-MCP1-Rv | ATGCATGTTTGGTGCATTAATACCA |
| Scrnseq-MCP2-Fw | CATTCAGTTTTAGGTTATGGGAACAT |
| Scrn-MCP2-MidRv | AACATTTCTATCACCGTCAATGAATTC |
| Seq-MCP2-Rv | TTCAGCCTGCTCTTTACCAA |
| Scrnseq-Sfp-Fw | GACGTGAAGAGAATCATTGTACCAGA |
| Scrn-Sfp-MidRv | GCAGCTTTTTCTTCGTCAGGAATTC |
| Seq-Sfp-Rv | TTCATCAGCTTGACTGGTAATGT |
| Scrnseq-MHP-Fw | GAATTGGTAAAGAGCAGGCTGA |
| Scrn-MHP-MidFw | GCTTTAGTAGAAACAGTTTGAGGAATTCG |
| Seq-MHP-Rv | GGCTTGTGCATCCTCATCAT |
| Scrnseq-MCP3-Fw | ACTGTTTCTACTAAAGCAAACGTTG |
| Scrn-MCP3-MidRv | TGATTAACGGTAAGCGTGAATTCA |

| | |
|---|---|
| Seq-MCP3-Rv | AACAGTTCTGGTAGGCTCCTT |
| Scrnseq-HTC1-Fw | ACGGAACTTACGCTGATGT |
| Scrn-HTC1-MidRv | CGGTACTTCTTTAATGTCAGGAATTC |
| Seq-HTC1-Rv | CTTCTTGTGAGTAGGACTGCAT |
| Scrnseq-HTC2-Fw | TTGCTTATGGCTATCACTGATGA |
| Scrn-HTC2-MidRv | CCCTTTTTTAATATCTCCAAAGAATTCA |
| Seq-HTC2-Rv | GGATTCGTTTGAACAATTCGTCA |
| Scrnseq-Tap-Fw | GTAATCGAAAAACGCACTCGTG |
| Scrn-Tap-MidRv | CTTTAAAGATGCTGCTTTATCCAGAATTC |
| Seq-Tap-Rv | ACAAAAGGTTGAGCAGATTGA |
| Scrnseq-Ttp-Fw | TGATTTAGGCGAATGGGTTGA |
| Scrn-Ttp-MidRv | GGTATACCCCAAAGCTCATCA |
| Seq-Ttp-Rv | TGTATTTGTTGTTGTATCGTCCA |
| Scrnseq-MTP-Fw | CACTTCAAATATGACAGCGAATATGCAG |
| Scrn-MTP-MidFw | TTGATGATGCAGCAATTATGAATTCA |
| Seq-MTP-Rv | CAATGTTAGCCATTTCTAGTTCAGGA |
| Scrnseq-gpG-Fw | GGAAACTTGCATTCCAAACAGA |
| Scrn-gpG-MidRv | CGATTTCCTAAAAATAATACATTCGATAGAATTC |
| Seq-gpG-Rv | TTCGCATTCATCAATAAAATCATCAA |
| Scrnseq-gpT-Fw | TCTCTTATGAACCTAACTCAGAAGATGCG |
| Scrn-gpT-MidRv | CATTTTTGCTTTGATTAACTTTCCGGATCC |
| Seq-gpT-Rv | AAGTTTTGAACTTGTTTAGCAGCGTC |
| Scrnseq-gpG*fs*T-Fw | ACTTGCTGTTAAAATCATTCCTGAACT |
| Scrn-gpG*fs*T-MidRv | ACTGAAAGCAATACTGGTAAAGCTG |
| Seq-gpG*fs*T-Rv | TTCTCTGGGCTTTCGATTCCTA |

**Recombinant protein primers**[ǂ, #]

| | |
|---|---|
| pNZ-PortalTuc-Fw | AGCAGCCCATGGTGAATTTGAAACCAATAAA |
| pNZ-PotalTuc-Rv | AGCAGCACTAGTTTA**ATGGTGATGGTGATGGTG**CCCCTCATTTGTTTCATTCTC |
| pNZ-MCP1Tuc-Fw | AGCAGCCCATGGATTCATCTGATTACTGGAG |
| pNZ-MCP1Tuc-Rv | AGCAGCACTAGTTTA**GTGATGGTGATGGTGATG**TCCTTTTCTTTTATTGC |
| pTX-MCP2Tuc-Fw | AGCAGCGGATCCATGGCTAAAGATGATTTCTT |
| pTX-MCP2Tuc-Rv | AGCAGCACTAGTTTA**GTGATGGTGATGGTGATG**AGTTCCTGGTATCCAGTCTTTT |
| pNZ-SfpTuc-Fw | AGCAGCCCATGGAACAAACAGAACTTTT |
| pNZ-SfpTuc-Rv | AGCAGCACTAGTTTA**GTGATGGTGATGGTGATG**ATAGCCTCCTGTTAATTTT |
| pTX-MHPTuc-Fw | AGCAGCCCATGGCAAAACAAAAAACAACACTTAC |
| pTX-MHPTuc-Rv | AGCAGCACTAGTTTA**GTGATGGTGATGGTGATG**TACACCCGTAAATGTGATATTA |
| pTX-MCP3Tuc-Fw | AGCAGCGGATCCATGGGGCGGCTACTAAGTCG |
| pTX-MCP3Tuc-Rv | AGCAGCACTAGTTTA**GTGATGGTGATGGTGATG**TAAGTGATAGCCATAAGCAA |

| | |
|---|---|
| pTX-HTC1Tuc-Fw | AGCAGCGGATCCATGGCTATCACTTATGAAAT |
| pTX-HTC1Tuc-Rv | AGCAGCACTAGTTTA**GTGATGGTGATGGTGATG**ATACAATCTGAACCTCCCTATCTTC |
| pTX-TapTuc-Fw | AGCAGCGGATCCATGAAAATAACTGGAATTGA |
| pTX-TapTuc-Rv | AGCAGCACTAGTTTA**GTGATGGTGATGGTGATG**TTTCGTCAACCTTTCTAA |

* Phosphorothioate linkages of recombineering oligos.

Ψ Nucleotides of recombineering oligos which are designed to introduce or alter the base sequence of TP901-1*erm* are underlined once, while introduced restriction enzyme sites are double underlined.

¥ Nucleotides of screening oligos which bind to mutated bases are underlined.

ǂ Restriction enzymes sites of recombinant protein construct oligos are double underlined.

# Hexahistidine tags are indicated in bold.
